# CAFs-derived LAM332 promotes CTCs formation and survival via ITGA3 and contributes to the metastasis of pancreatic ductal adenocarcinoma

**DOI:** 10.1038/s41419-026-08642-z

**Published:** 2026-03-25

**Authors:** Haodong Tang, Wenyuan Shi, Siyuan Tan, Zheng Zhang, Qiannan Zhang, Pengcheng Zhou, Yang Wang, Zhengqing Lei, Fangfang Hu, Shan Gao, Jiahua Zhou

**Affiliations:** 1 Department of Surgery, School of Medicine, Southeast University, Nanjing, Jiangsu China; 2 Department of General Surgery, Affiliated Hospital of Nantong University, Nantong, Jiangsu China; 3 Department of Hepato-Pancreatico-Biliary Surgery, Zhongda Hospital Southeast University, Nanjing, Jiangsu China; 4 Zhongda Hospital, School of Life Sciences and Technology, Advanced Institute for Life and Health, Southeast University, Nanjing, China

**Keywords:** Metastasis, Cancer genetics

## Abstract

Metastasis remains the primary cause of mortality in pancreatic ductal adenocarcinoma (PDAC). Circulating tumor cells (CTCs) are key players in metastasis, yet the mechanisms governing CTCs formation and survival are incompletely understood. Here, we identify ITGA3 as a key driver of CTCs generation and metastatic progression in PDAC. Integrated proteomic and transcriptomic analyses, coupled with clinical specimen validation, revealed that ITGA3 expression positively correlates with CTCs abundance and poor prognosis. Mechanistically, ITGA3 promotes epithelial–mesenchymal transition (EMT), enhances matrix metalloproteinase expression, and facilitates tumor cell detachment, thereby initiating CTCs formation. Importantly, cancer-associated fibroblasts (CAFs) secrete laminin-332 (LAM332), which engages ITGA3 on PDAC cells to promote proliferation and invasion, drive homotypic CTC clustering, and suppress apoptosis, collectively sustaining CTCs' survival. Neutralization of CAFs-derived LAM332 impaired tumor cell proliferation and invasion, disrupted CTC cluster formation, increased apoptosis, reduced hepatic and pulmonary metastasis, and prolonged survival in mouse models. These findings uncover a CAFs–LAM332–ITGA3 axis that orchestrates CTCs formation and survival, and highlight this stromal–tumor interaction as a promising therapeutic target to mitigate metastatic progression in PDAC.

## Introduction

Pancreatic ductal adenocarcinoma (PDAC) is an aggressive gastrointestinal malignancy with an exceptionally poor prognosis [1, 2]. More than half of patients present with advanced disease and distant metastases, most frequently to the liver, lungs, and lymph nodes [3]. Metastasis is a multistep, tightly regulated process encompassing local invasion, intravasation into blood or lymphatic circulation, survival during transit, extravasation, and colonization at secondary sites [4]. Circulating tumor cells (CTCs) are shed from primary or metastatic lesions into the circulation as precursors of metastases [5]. CTCs are detected more frequently in patients with metastatic disease than in those with localized tumors, and their presence correlates strongly with poor outcomes in PDAC [6]. Processes such as epithelial–mesenchymal transition (EMT), extracellular matrix (ECM) remodeling, and hypoxia have been implicated in CTCs generation and metastatic spread [7–9]. Yet, the molecular mechanisms underlying CTC formation in PDAC remain poorly understood. CTCs encounter hostile conditions, including oxidative stress, immune surveillance, and hemodynamic shear forces in the bloodstream [10]. Only a small fraction withstands these challenges to form metastatic lesions [11]. Evidence from other cancer types suggests that homotypic CTC clustering enhances survival in circulation and confers greater metastatic potential, but the molecular determinants of these processes in PDAC are largely unexplored [12].

Integrin signaling is linked to CTCs' biology in other cancers. For example, inhibition of the integrin αv/FAK axis reduces CTC cluster formation in oral squamous cell carcinoma [13], while targeting the integrin β1/Src pathway suppresses CTC formation and increases apoptotic susceptibility in lung cancer [14]. Integrin subunit alpha-3 (ITGA3), a transmembrane glycoprotein of the integrin family, mediates cell-ECM adhesion and activates intracellular pathways that regulate stemness, proliferation, therapeutic resistance, and metastasis in PDAC [15, 16]. The ECM, a major ligand source for integrins, further shapes CTCs' behavior. For example, Hsp4-induced collagen deposition promotes platelet–CTC cluster formation via integrin α2β1 signaling [7]. In PDAC, cancer-associated fibroblasts (CAFs) are the primary source of ECM proteins, engaging in bidirectional integrin-mediated interactions, contributing to matrix remodeling, oncogenic signaling, and metastatic dissemination [17, 18]. Despite these insights, the functional roles of integrin and ECM components in CTCs formation and survival in PDAC have yet to be fully elucidated.

In this study, we identify ITGA3 as a critical regulator of CTCs formation, survival, and metastasis in PDAC. We further show that CAFs-derived laminin-332 (LAM332) induces EMT and promotes homotypic CTC clustering and survival via ITGA3-dependent signaling, revealing a previously unrecognized CAFs–integrin axis that fuels metastatic progression in PDAC.

## Materials and methods

### Clinical samples

Tissue samples were obtained from 60 PDAC patients at Zhongda Hospital, Southeast University, following surgical resection. Patients had not undergone prior chemotherapy or radiotherapy. Samples were frozen in liquid nitrogen or fixed in formalin and embedded in paraffin. Informed consent was obtained, and the study was approved by the Ethics Committee (2016ZDSYLL027.0). Pathological evaluation and subsequent analyses were performed by investigators blinded to clinical data and outcomes.

### Label-free quantitative proteomics

Formalin-fixed paraffin-embedded (FFPE) tumor tissue samples were collected from 10 patients with intravascular tumor cells (CTC group) and 10 patients without intravascular tumor cells (no CTC group). These 20 FFPE tumor tissue samples were obtained from the tissue bank of the Pathology Department, Zhongda Hospital. All samples were subjected to 4D label-free quantitative proteomic analysis, which was performed by GeneChem Co., Ltd. (Shanghai, China). Differentially expressed proteins (DEPs) between the CTC and no CTC groups were identified based on an |FC| > 1.5 and a false discovery rate (FDR) < 0.05.

### Cell culture

Pancreatic cancer cell lines (PANC-1, CAPAN-2, BXPC-3, MIAPACA-2, CFPAC-1) and normal pancreatic ductal epithelial (HPNE) cells were sourced from the Shanghai Cell Bank of the Chinese Academy of Sciences (China). Normal fibroblasts (NFs) and immortalized cancer-associated fibroblasts (CAFs) were kindly provided by Professor Zhang [19]. Pancreatic cancer cell lines and HPNE were cultured in DMEM (GIBCO, USA) with 10% FBS. NFs and CAFs were cultured in specialized medium for human fibroblasts (iCell-002a-001b, iCell). All cells were incubated at 37 °C in a 5% CO_2_ atmosphere.

### Quantitative real-time PCR (qRT‒PCR)

Total RNA was extracted using TRIzol (Invitrogen, USA) and cDNA synthesized using the SweScript RT I Kit (Servivebio, China). qRT-PCR was performed with SYBR Green Master Mix (Servivebio, China), and gene expression was analyzed by the 2^-ΔΔCt method. The sequences of the primers used in the study are provided in Supplementary Table 1.

### Western blotting

Total protein was extracted using RIPA buffer (Servicebio, China), and protein concentration was measured by BCA assay (Beyotime, China). Equal amounts of protein (30 μg/well) were separated by SDS-PAGE (Epizyme, China) and transferred to PVDF membranes (Millipore, Germany). Membranes were blocked with 5% skim milk for 2 h, incubated with primary and secondary antibodies (Supplementary Table 2), and signals were detected using BeyoECL Star (Beyotime, China).

### Enzyme-linked immunosorbent assay (ELISA)

Serum from 20 PDAC patients and 20 healthy controls, as well as conditioned medium from cultured cells, were analyzed for LAM332 concentration using an ELISA kit (Mlbio, China). For this calculation, the absorbance (optical density, OD) of each well was measured at 450 nm, and a linear regression was calculated using the concentrations and OD values of the standard products to determine the LAM332 concentrations for each group on the basis of the OD values of the samples.

### Small interfering RNAs (siRNAs) and lentiviral vectors

siRNAs targeting ITGA3 and negative controls (Supplementary Table 3) were synthesized by GenePharma (Shanghai, China). Transfections were carried out using siRNA-mate (GenePharma, Shanghai, China). Lentiviral particles for ITGA3/ITGB1 shRNA or overexpression were generated by GenePharma (Shanghai, China), and stable cell lines were selected using puromycin.

### Cell counting kit-8 (CCK-8) assay

Cells (1000/well) were seeded in a 96-well plate and treated with 10 μL of CCK-8 solution (Beyotime, China) and 100 μL of fresh medium. After 2 h of incubation at 37 °C, absorbance at 450 nm was measured using a BioTek ELx800 microplate reader.

### Colony formation assay

Cells were seeded at a density of 1000 cells per well in 6-well plates and cultured for 2 weeks. Afterward, the cells were fixed with 4% paraformaldehyde and stained with 0.5% crystal violet for colony quantification.

### 5-Ethynyl-2′-deoxyuridine (EdU) assay

Cell proliferation was measured using the BeyoClick™ EDU-488 Cell Proliferation Detection Kit (Beyotime, China). Cells (1 × 10⁵/well) were seeded in 24-well plates, cultured, and subjected to EdU labeling, fixation, washing, and nuclear staining. Green fluorescence intensity and cell numbers were observed using a fluorescence microscope.

### Wound healing assay

Cells were seeded in 6-well plates and grown to 80–90% confluence. A scratch was made using a plastic pipette tip, and wound edges were marked and photographed at 0 and 48 h. Cell migration distance and wound closure percentage were quantified using ImageJ (NIH, USA).

### Transwell migration and invasion assays

For migration, 4 × 10⁴ cells in serum-free medium were seeded in transwell plates (8 μm pore size; Falcon, USA), with DMEM containing 10% FBS in the lower chamber. After 48 h of incubation, cells were fixed with formaldehyde, stained with crystal violet, and the cells on the lower surface were counted. For the invasion experiment, cells were seeded in transwell plates coated with Matrigel matrix (Corning, USA). The other experimental procedures were the same as those used in the migration experiment.

### Suspension culture

Ultralow attachment plates were coated with 30 mg/mL poly(2-hydroxyethylmethacrylate) (HEMA) (Sigma‒Aldrich, USA). After drying overnight, the plates were irradiated with UV light for 30 min, washed with PBS three times, and cells were seeded for subsequent experiments.

### Cell clustering assay

Pancreatic cancer cells (2.5 × 10⁴/well) were seeded in poly-HEMA-coated 6-well plates. Cell morphology was observed at 6 and 24 h using a Leitz microscope (10× magnification). Clustering capability was assessed by measuring the size of cell aggregates with ImageJ.

### Live/dead viability assay

Cells were incubated with 1 μM calcein AM and 1 μM PI (Beyotime, China) for 30 min at room temperature. Live (green) and dead (red) cells were observed using a fluorescence microscope.

### Apoptosis assay

Suspended cells were centrifuged at 800 × *g* for 5 min, washed with PBS, and resuspended in 500 μL binding buffer. The cells were incubated with 5 μL of Annexin V and 5 μL of PI (Beyotime, China) for 5–10 min at room temperature. Flow cytometry was performed to analyze the staining.

### Cell adhesion assay

Laminin, collagen, and fibronectin (Sigma, USA) were coated on 96-well plates at 37 °C for 2 h, washed with PBS, and blocked with 2% BSA at 37 °C for 1 h. Cells (1 × 10⁴/well) were resuspended in serum-free DMEM and incubated for 30 min at 37 °C with 5% CO_2_. Adherent cells were fixed with formaldehyde, stained with crystal violet, and the absorbance at 560 nm was measured. Alternatively, cells were stained with DAPI and imaged using a fluorescence microscope, and cell counting was performed using ImageJ.

### Coculture of CAFs and cancer cells

Tumor cells (5 × 10⁴/well) were seeded in six-well plates and cultured in a humidified chamber with 5% CO_2_ at 37 °C. After 2 days, the tumor cell medium was replaced with fresh medium from CAFs (5 × 10⁴/well), and incubation continued for 24 h before further experiments.

### Immunohistochemical staining

FFPE tissue sections were deparaffinized, rehydrated, and subjected to antigen retrieval in sodium citrate (pH 6.0) via microwave. After blocking with 3% BSA, sections were incubated with primary antibodies overnight at 4 °C, followed by secondary antibodies (Supplementary Table 2). Immunostaining was performed with DAB, and nuclei were counterstained with hematoxylin. ITGA3 expression was scored semiquantitatively using the immunoreactivity score (IRS), based on staining intensity and the percentage of positive cells. Patients were classified into high-expression (IRS > mean score) and low-expression (IRS ≤ mean score) groups.

### Immunofluorescence staining

FFPE tissue sections were deparaffinized, rehydrated, and subjected to antigen retrieval with EDTA buffer (pH 9.0). After treatment with 3% hydrogen peroxide, sections were blocked with BSA and incubated with primary (Supplementary Table 2). For immunofluorescence, α-SMA, LAM332, vimentin, plectin, and ITGA3 were labeled with CY3 (red), FITC (green), Cy5 (pink), and respective combinations. Nuclei were counterstained with DAPI. Sections were imaged using a scanner, and data were processed with CaseViewer software.

### Animal experiments

All animal procedures were approved by the Institutional Animal Care and Use Committee of Southeast University (20230306002). Five-week-old male/female BALB/c nude mice (Huachuang Sino Co., Ltd.) were used for subcutaneous xenograft and orthotopic models. Animals were randomly allocated to experimental groups. For subcutaneous xenografts (*n* = 5 mice/group), 1 × 10^6^ tumor cells in PBS/Matrigel (1:1) were injected into the right axilla. Tumor volume was measured weekly. For orthotopic metastasis (*n* = 5 mice/group), 1 × 10^6^ tumor cells in PBS/Matrigel (1:1) were injected into the pancreas. In the CAFs group, CAFs and tumor cells were co-injected (1:1). LAM332 protein/neutralizing antibody (20 mg/kg) was administered intraperitoneally every 3 days. Tumor progression was monitored using the IVIS® Spectrum imaging system. Blood samples were processed for CTCs identification using the homoporous polydimethylsiloxane membrane microfilter [20]. Liver and lung metastases were analyzed via H&E staining. Survival was assessed in a separate cohort of mice (*n* = 10 mice/group). Investigators responsible for data collection and analysis were blinded to group allocation throughout the experiments.

### Metastatic burden

Genomic DNA was extracted from the blood, liver, and lung tissues of nude mice using the Genomic DNA Extraction Kit (Beyotime, China). Real-time PCR was conducted to quantify reporter gene (puromycin R) and vimentin expression. Tumor cell abundance in the blood, liver, and lungs was assessed to evaluate metastatic spread. The relative metastatic burden was calculated as 10,000 × 2^−ΔCt, where ΔCt is the difference in Ct values between vimentin and the reporter gene [21].

### Data acquisition

The datasets GSE144561, GSE28735, GSE62452, GSE71729, and GSE154778 were retrieved from the Gene Expression Omnibus (GEO) database (https://www.ncbi.nlm.nih.gov/geo/). The PAAD_CRA001160 dataset was obtained from the Tumor Immune Single-cell Hub 2 (TISCH2) database (http://tisch.comp-genomics.org/home/). Additionally, PDC000248 TMT data were downloaded from the Clinical Proteomic Tumor Analysis Consortium(CPTAC) database (https://cptac-data-portal.georgetown.edu/).

### Statistical analysis

All analyses were performed using R (version 4.1) and GraphPad Prism (version 8.0). GSE28735 and GSE62452 were normalized with the “SVA” package and validated by PCA. Candidate gene coefficients were obtained via the LASSO algorithm. Differential expression was analyzed using “Limma” (*p* < 0.05, |log2FC| > 1). Single-cell RNA-seq data were processed with “Seurat,” and GO enrichment was performed using “clusterProfiler” (*P* < 0.05). Statistical comparisons were made using Student’s *t*-test or one-way ANOVA. Correlations were assessed with Pearson’s coefficient. Survival analysis was conducted with Kaplan–Meier and log-rank test. Univariate and multivariate Cox regression identified prognostic factors. *P* < 0.05 was considered statistically significant. All experiments were repeated at least three times.

## Results

### The identification of ITGA3 as a potential regulator of CTCs formation and metastasis in PDAC

**Fig. 1:**
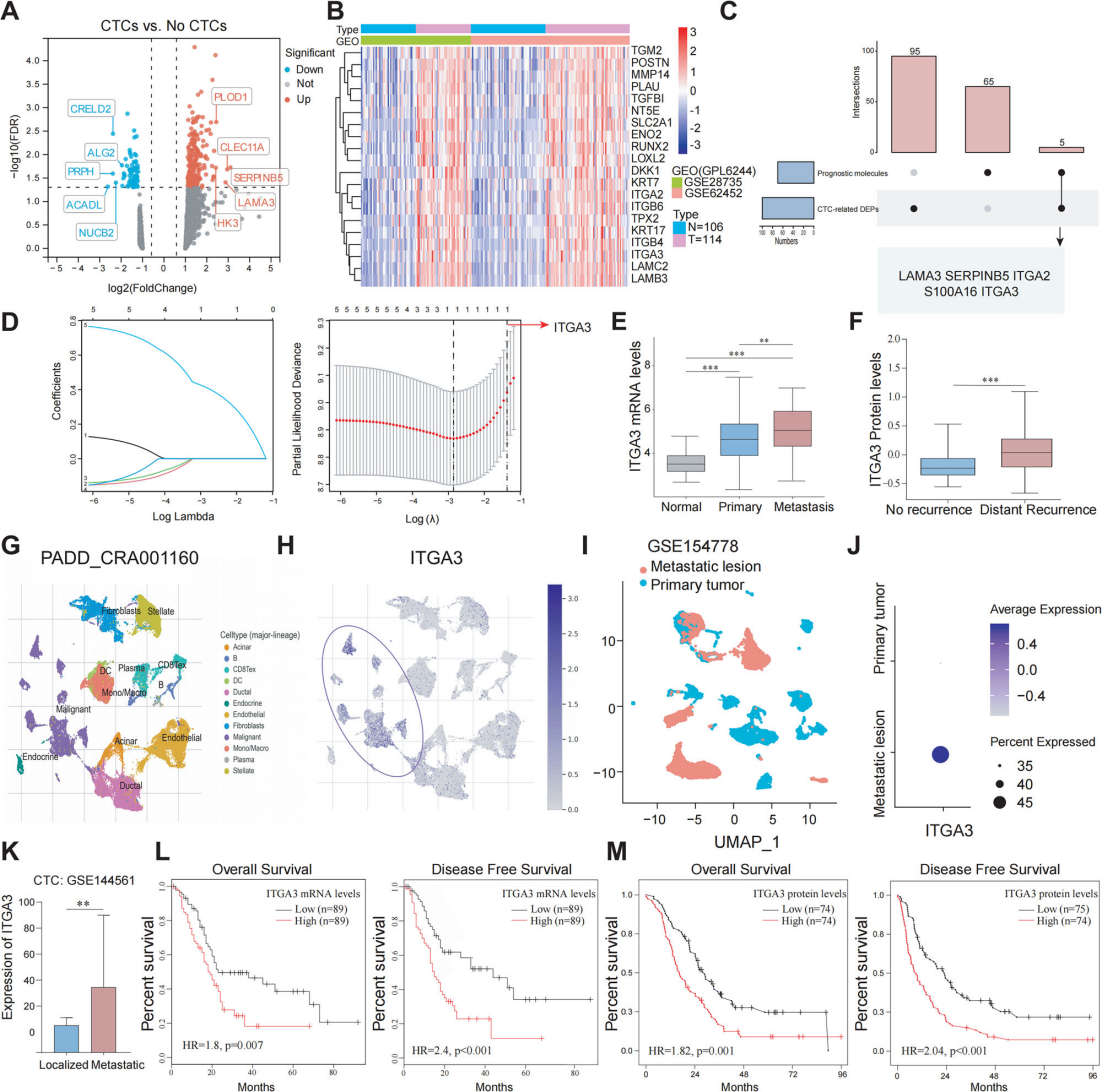
The Identification of ITGA3 as a potential regulator of CTCs Formation and Metastasis in PDAC. **A** Volcano plot of DEPs between the CTC (*n* = 10) and the no CTC groups (*n* = 10). **B** Heatmaps of DEGs between normal samples (*n* = 106) and PDAC samples (*n* = 114). **C** Venn diagram of 100 CTC-DEPs and 70 prognostic molecules. **D** Tenfold cross-validation was performed with adjustments for parameter selection in the LASSO regression and LASSO coefficient plots for the 5 candidate genes. **E** Differences in ITGA3 mRNA expression in groups (*n* = 61 for normal pancreases, *n* = 145 for primary PDAC, *n* = 42 for Met liver/lung/lymph node). **F** Differences in ITGA3 protein expression in groups (*n* = 29 for no recurrence, *n* = 111 for distant recurrence). **G** Cell clustering annotations of the UMAP plot in the PAAD_CRA001160 dataset (*n* = 24 for primary tumor, *n* = 11 for pancreases). **H** UMAP plots of ITGA3 expression in the PAAD_CRA001160 dataset. **I** UMAP plots of cells from primary tumor tissues (*n* = 10) and metastatic lesions (*n* = 6). **J** Dot plot showing the average expression level of ITGA3 in primary tumors and metastatic lesions. **K** Expression of ITGA3 in CTC samples from patients with localized PDAC (*n* = 42) and those with metastatic PDAC (*n* = 18). **L**, **M** Association of ITGA3 mRNA and protein expression with overall survival and disease-free survival in patients with PDAC, respectively.

To identify key regulators of CTCs formation and subsequent metastasis in PDAC, we first performed comparative proteomic profiling of patients with detectable CTCs (*n* = 10) and those without CTCs (*n* = 10). This analysis identified 100 differentially expressed proteins (DEPs), including 70 upregulated and 30 downregulated proteins in the CTC group (Fig. 1A; Supplementary Table 4). To further narrow down clinically relevant CTC-related molecules, we integrated RNA-seq data from the GSE28735 and GSE62452 datasets and performed batch effect correction (Supplementary Fig. 1A). Differential expression analysis identified 332 differentially expressed genes (DEGs) between PDAC and normal pancreatic tissues (Fig. 1B). Subsequently, univariate Cox regression analysis of these DEGs revealed 70 genes significantly associated with overall survival (*p* < 0.05), which were defined as candidate prognostic genes (Supplementary Table 5). Intersecting these 70 prognosis-related DEGs with the 100 CTC-related DEPs yielded five candidate molecules (Fig. 1C). Finally, least absolute shrinkage and selection operator (LASSO) regression analysis of these candidate genes identified ITGA3 as the most critical prognostic biomarker associated with CTCs (Fig. 1D). Multi-cohort validation consistently confirmed the prognostic and metastatic significance of ITGA3. In the GSE71729 dataset, ITGA3 expression was significantly higher in metastatic PDAC compared with primary tumors and normal tissues (Fig. 1E). Similarly, CPTAC proteomic data demonstrated elevated ITGA3 protein levels in patients with distant recurrence (Fig. 1F). Single-cell RNA-seq (PAAD_CRA001160) further revealed that ITGA3 was predominantly expressed in malignant epithelial subpopulations (Fig. 1G, H; Supplementary Fig. 1B, C) and was markedly enriched in metastatic lesions compared with primary tumors (Fig. 1I, J; Supplementary Fig. 1D, E). Moreover, CTCs isolated from metastatic PDAC patients displayed significantly higher ITGA3 expression compared with those from localized disease (Fig. 1K). Clinically, Kaplan–Meier survival analysis demonstrated that high-ITGA3 mRNA levels were associated with worse overall survival (OS) and disease-free survival (DFS) (Fig. 1L), and these findings were corroborated at the protein level in CPTAC data (Fig. 1M). Collectively, these results strongly support ITGA3 as a CTCs- and metastasis-related factor with potential prognostic and therapeutic relevance in PDAC.

### Clinical significance of ITGA3 expression in relation to CTCs formation, tumor invasion, and prognosis in PDAC patients

**Fig. 2:**
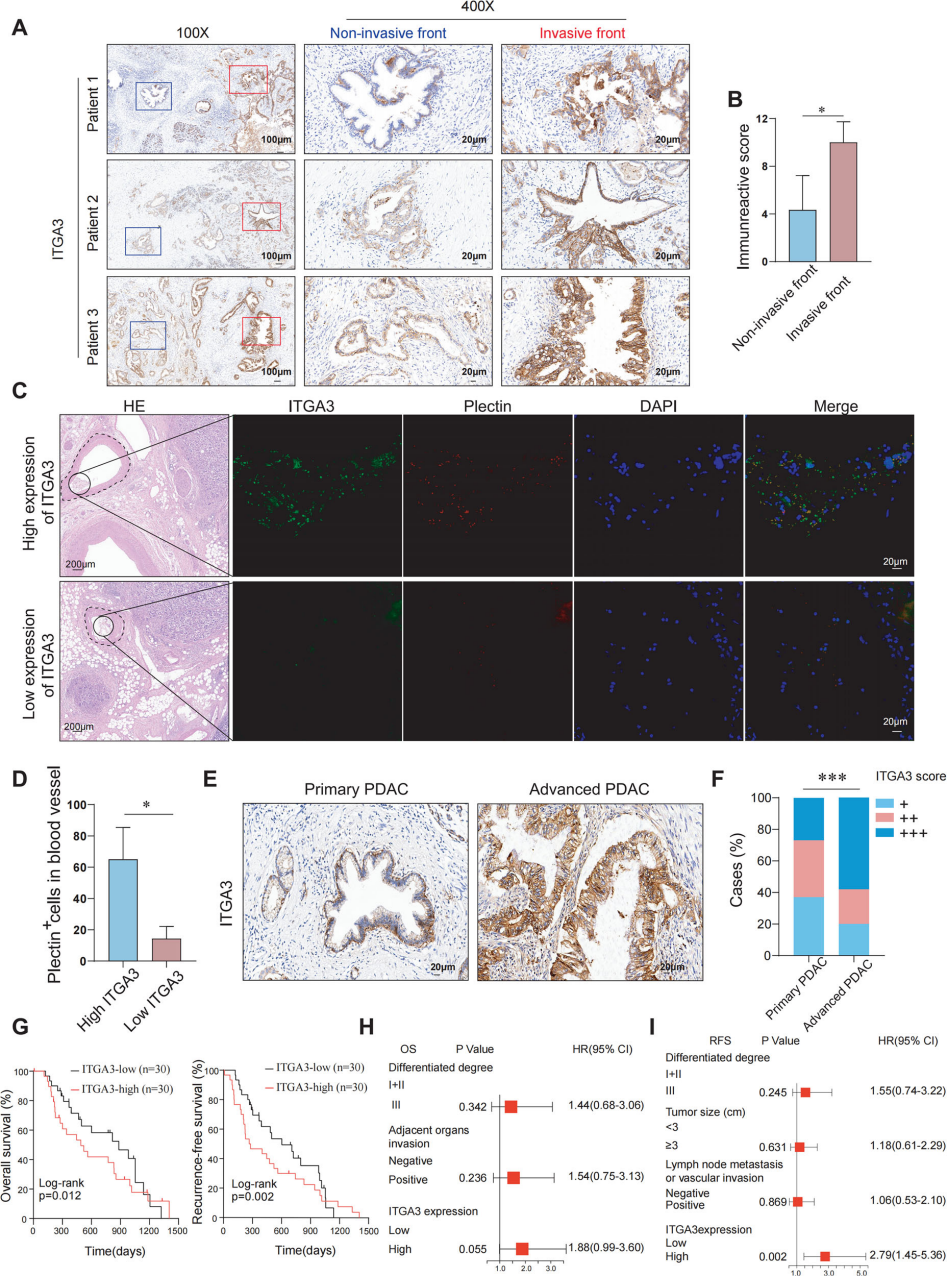
Clinical significance of ITGA3 expression in relation to CTCs formation, tumor invasion, and prognosis in PDAC patients. **A** Representative images of IHC for ITGA3 in the invasive front and noninvasive front of the tumor. **B** Immunoreactive scores (IRS) were quantified for comparison between the two groups (*n* = 10 for noninvasive front, *n* = 10 for invasive front). **C** Multiplex immunofluorescence images showing the expression of ITGA3 and the detection of CTCs in the peritumoral vasculature. **D** Comparison of CTC counts in patients with different ITGA3 expression levels (plectin as a biomarker of CTCs in PDAC) (*n* = 10 for high ITGA3, *n* = 10 for low ITGA3). **E** Representative images of IHC for ITGA3 in primary PDAC and advanced PDAC. **F** The frequency of cases with the indicated scores of ITGA3 immunoreactivity (*n* = 36 for advanced PDAC, *n* = 24 for primary PDAC). **G** Kaplan‒Meier survival curves showing the correlation between the ITGA3 expression level and survival (OS and RFS) (*n* = 30 for ITGA3-high, *n* = 30 for ITGA3-low). **H**, **I** Forest plot of multivariate Cox regression analysis of OS and RFS in 60 patients with PDAC.

Given that the tumor invasive front (TIF) often harbors the most aggressive cancer cell subpopulations and more accurately reflects metastatic potential than other tumor regions [22], we next assessed ITGA3 expression in this compartment of PDAC tissues. In our cohort, ITGA3 expression was significantly higher at the TIF compared with the tumor interior (Fig. 2A, B). To further examine the relationship between ITGA3 and CTCs formation, we performed tissue immunofluorescence after identifying vasculature by HE staining. Patients with high-ITGA3 expression exhibited a markedly greater number of CTCs (plectin-positive cells) (Fig. 2C, D). Consistently, advanced-stage PDAC tissues also showed significantly elevated ITGA3 expression (Fig. 2E, F). Clinicopathological correlation analysis revealed that higher ITGA3 immunohistochemical scores were positively associated with larger tumor size, poor differentiation, adjacent tissue invasion, and lymphovascular invasion (Table 1). Survival analysis further demonstrated that patients in the high-ITGA3 group had significantly worse OS and relapse-free survival (RFS) compared with those in the low-ITGA3 group (Fig. 2G). Moreover, multivariate Cox regression confirmed ITGA3 expression as an independent predictor of RFS (Fig. 2H, I; Supplementary Table 6). Together, these results indicate that ITGA3 is enriched at the invasive tumor front, promotes CTCs generation, correlates with aggressive pathological features, and serves as an independent prognostic biomarker in PDAC.

**Table 1:** Clinical baseline characteristics of PDAC patients with high or low-ITGA3 expression.

Variable	Overall, *N* = 60	High, *N* = 30	Low, *N* = 30	*p*-value
Gender				0.596
Female	23 (38%)	13 (43%)	10 (33%)	
Male	37 (62%)	17 (57%)	20 (67%)	
Age				0.999
>65	31 (52%)	15 (50%)	16 (53%)	
≤65	29 (48%)	15 (50%)	14 (47%)	
Tumor site				0.568
body/tail	17 (28%)	7 (23%)	10 (33%)	
Head	43 (72%)	23 (77%)	20 (67%)	
Tumor size (cm)				0.029
<3	21 (35%)	6 (20%)	15 (50%)	
≥3	39 (65%)	24 (80%)	15 (50%)	
Differentiated degree				0.021
I + II	48 (80%)	20 (67%)	28 (93%)	
III	12 (20%)	10 (33%)	2 (6.7%)	
Adjacent organs invasion				0.025
Negative	19 (32%)	5 (17%)	14 (47%)	
Positive	41 (68%)	25 (83%)	16 (53%)	
Lymph node metastasis or vascular invasion				0.003
Negative	24 (40%)	6 (20%)	18 (60%)	
Positive	36 (60%)	24 (80%)	12 (40%)	

### ITGA3 promotes the proliferation and migration of PDAC cells

**Fig. 3:**
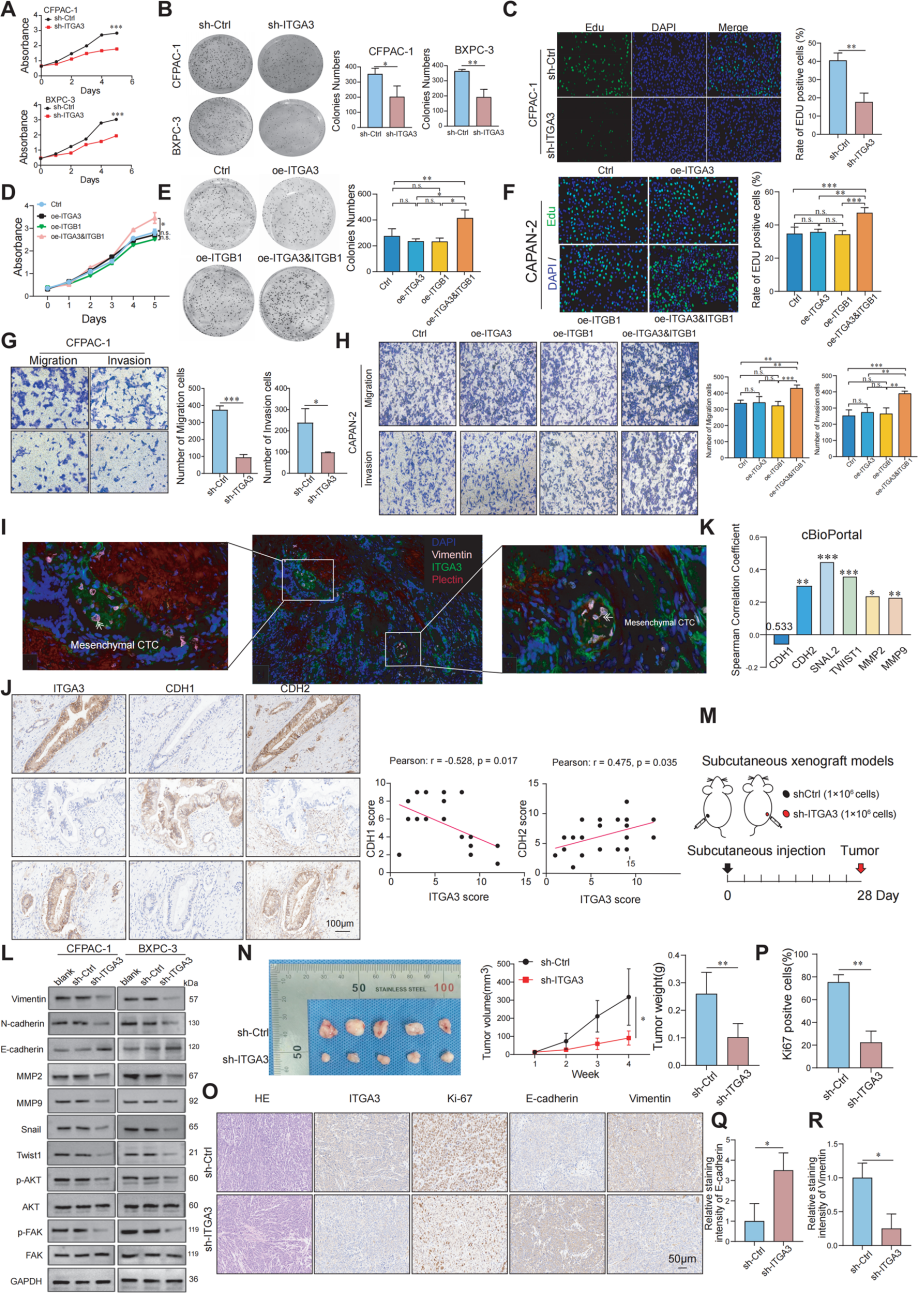
ITGA3 promotes the proliferation and migration of PDAC cells. **A** PDAC cells were harvested for the CCK-8 cell proliferation assay, **B** colony formation assay, and **C** EdU assay. **D** CAPAN-2 cells (Ctrl, oe-ITGA3, oe-ITGB1, and oe-ITGA3&ITGB1) were harvested for the CCK-8 cell proliferation assay, **E** colony formation assay, and **F** EdU assay (*n* = 3 independent experiments). **G** Cell migration and invasion capacity were determined via a transwell assay (*n* = 3 independent experiments). **H** The cell migration and invasion capacities of CAPAN-2 cells (Ctrl, oe-ITGA3, oe-ITGB1, and oe-ITGA3&ITGB1) were determined via a transwell assay (*n* = 3 independent experiments). **I** Multiplex immunofluorescence images displaying the expression of ITGA3 and vimentin in CTCs within the peritumoral vasculature. **J** The correlation between the immunoreactivity scores of ITGA3 and the immunoreactivity scores of CDH1 and CDH2 in PDAC tissues (*n* = 20 PDAC patients). **K** Cancer molecular expression profile data from cBioPortal revealed a correlation between ITGA3 and EMT-related molecules. **L** The expression levels of EMT-related proteins (Vimentin, N-cadherin, E-cadherin, Snail, and Twist1), matrix metalloproteinases (MMP2 and MMP9), and p-AKT and p-FAK were assessed via western blotting (*n* = 3 independent experiments). **M** Schematic diagram of the subcutaneous xenograft models. **N** Comparison of subcutaneous tumor volume and weight between the control group and the ITGA3-knockdown group (*n* = 5 mice/group). **O**‒**R** H&E and IHC staining of Ki67, E-cadherin, and vimentin are shown (*n* = 5 mice/group).

Since RNA-seq and clinical data revealed strong correlations between ITGA3 expression, CTC abundance, metastasis, and poor prognosis, we next investigated its functional role in PDAC cells. Two ITGA3-high cell lines (BXPC-3 and CFPAC-1) and one ITGA3-low cell line (CAPAN-2) were selected for in vitro studies (Supplementary Fig. 2A). ITGA3 was efficiently knocked down using siRNAs, with si-ITGA3#2 showing the strongest knockdown and subsequently used to establish stable shRNA-mediated ITGA3-knockdown cells (Supplementary Fig. 2B–E). Cell viability assays demonstrated that ITGA3 knockdown markedly suppressed proliferation in ITGA3-high lines (Fig. 3A–C; Supplementary Fig. 2F). In contrast, overexpression of ITGA3 alone in ITGA3-low CAPAN-2 cells did not enhance proliferation (Fig. 3D–F; Supplementary Fig. 2G). Because ITGA3 functions through dimerization with ITGB1 to form integrin α3β1, we hypothesized that single-gene overexpression might be insufficient. Supporting this notion, neither ITGA3 nor ITGB1 overexpression alone altered proliferation, whereas co-overexpression of both significantly promoted growth (Fig. 3D–F; Supplementary Fig. 2H). Similarly, knockdown of ITGA3 reduced migration and invasion, while co-overexpression of ITGA3 and ITGB1 markedly enhanced these processes (Fig. 3G, H; Supplementary Fig. 2I, J). Together, these results establish ITGA3 as a functional mediator of PDAC cell proliferation, migration, and invasion through the integrin α3β1 complex. Given that CTCs frequently acquire mesenchymal traits to facilitate dissemination, we next assessed whether ITGA3 contributed to epithelial–mesenchymal transition (EMT). In clinical samples, ITGA3-positive CTCs with a mesenchymal phenotype were readily observed within vasculature (Fig. 3I). Consistently, IHC revealed an inverse correlation between ITGA3 and CDH1 (E-cadherin) and a positive correlation with CDH2 (N-cadherin) (Fig. 3J), which was further corroborated by cBioPortal analysis (Fig. 3K). Functionally, ITGA3 knockdown decreased expression of invasion-associated proteins (MMP2, MMP9) and EMT regulators (Vimentin, N-cadherin, Snail, Twist1), while increasing E-cadherin levels (Fig. 3L). In parallel, phosphorylation of FAK and AKT was markedly suppressed (Fig. 3L). In vivo, ITGA3 knockdown significantly decreased tumor growth in subcutaneous xenograft models (Fig. 3M, N), accompanied by reduced Ki67 and vimentin expression and increased E-cadherin expression (Fig. 3O–R). Together, these findings demonstrate that ITGA3 promotes PDAC progression by enhancing proliferation, migration, and invasion through α3β1-mediated activation of FAK/AKT signaling, thereby driving EMT and mesenchymal-like CTCs generation that initiates metastasis.

### ITGA3 promotes the formation of homotypic CTC clusters and suppresses apoptosis

**Fig. 4:**
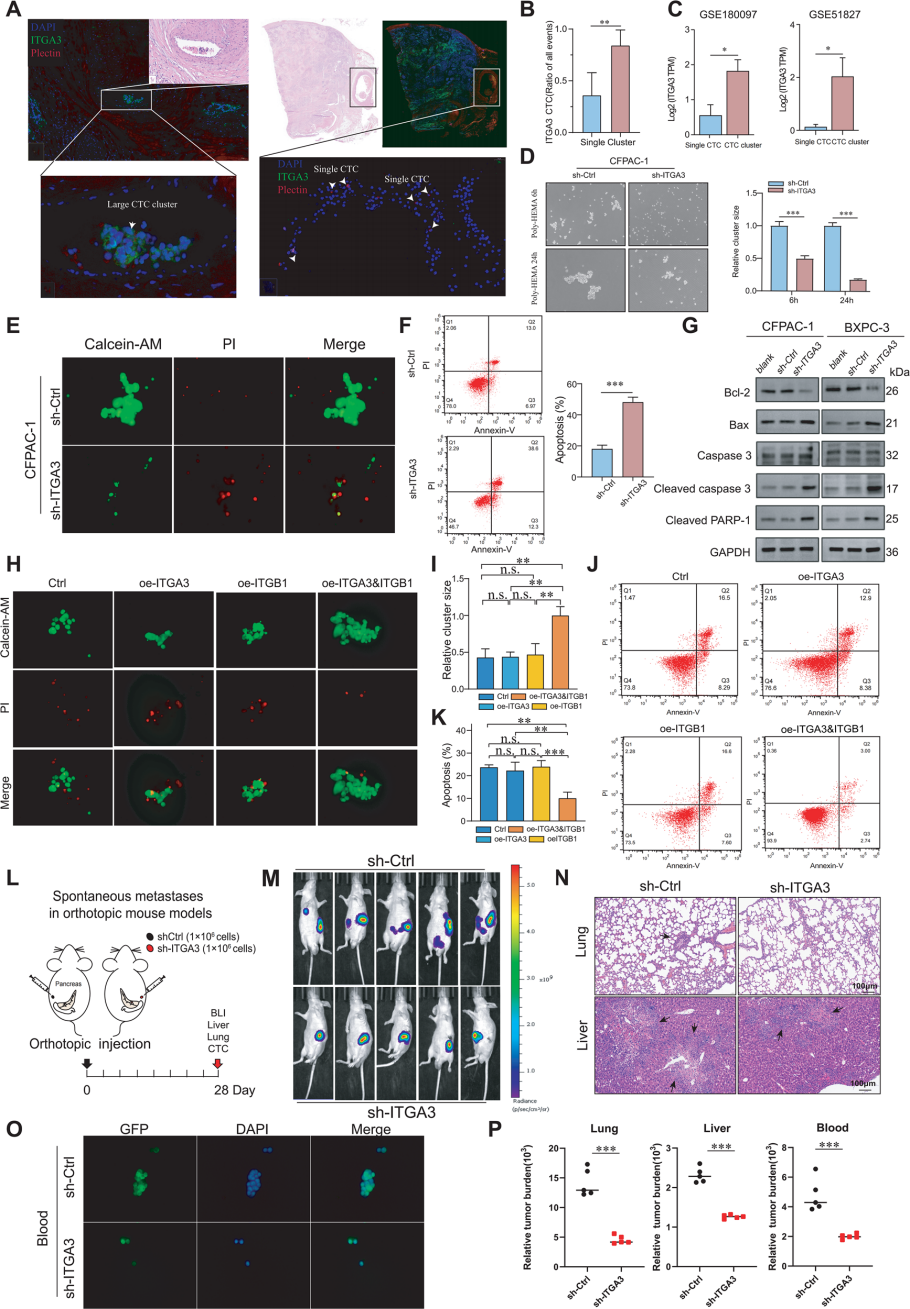
ITGA3 promotes the formation of homotypic CTC clusters and suppresses apoptosis. **A**, **B** Multiplex immunofluorescence images demonstrating the association between ITGA3 expression and CTC clusters in the peritumoral vasculature (*n* = 20 PDAC patients). **C** Analysis of the GEO database revealed significantly higher ITGA3 expression levels in CTC clusters than in single CTCs (GSE180097: *n* = 13 for single CTC, *n* = 17 for CTC cluster; GSE51827: *n* = 15 for single CTC, *n* = 14 for CTC cluster). **D** Cell clustering capacity was assessed in ITGA3-knockdown and control cells (*n* = 3 independent experiments). **E**, **F** Live/dead viability assays and apoptosis assays were performed after 24 h of cell suspension culture (*n* = 3 independent experiments). **G** Western blot analysis was used to evaluate the expression levels of apoptosis-related proteins in control and ITGA3-knockdown cells (*n* = 3 independent experiments). **H**–**K** Live/dead viability and apoptosis assays were performed after 24 h of culture with a suspension of CAPAN-2 (*n* = 3 independent experiments). **L** Schematic representation of spontaneous metastases in orthotopic mouse models. **M** Representative BLI images of mice 28 days after orthotopic injection of the indicated cells (*n* = 5 mice/group). **N** Representative H&E staining images of mouse lungs and livers. **O** Representative fluorescence images of GFP-positive CTCs in the peripheral blood of mice. **P** Metastatic burdens of liver, lung, and blood tissue at the endpoint (*n* = 5 mice/group).

Only a small fraction of CTCs survive the hostile circulatory environment to establish distant metastases, and prior studies have shown that homotypic CTC clusters confer a survival advantage [23]. To investigate whether ITGA3 contributes to this process, we first examined patient samples, where ITGA3⁺ cells were more frequently detected within CTC clusters than among single CTCs (Fig. 4A, B). Consistently, analysis of the GSE51827 and GSE180097 datasets confirmed higher ITGA3 expression in CTC clusters than in solitary CTC (Fig. 4C). In suspension cultures, ITGA3 knockdown markedly impaired cell cluster formation (Fig. 4D; Supplementary Fig. 3A) and increased single-cell death, as demonstrated by live/dead staining and flow cytometry (Fig. 4E, F; Supplementary Fig. 3B, C). Western blot analysis further showed reduced Bcl-2 and elevated Bax, cleaved caspase-3, and cleaved PARP-1 following ITGA3 knockdown (Fig. 4G). Conversely, co-overexpression of ITGA3 and ITGB1 in ITGA3-low CAPAN-2 cells enhanced both aggregation and resistance to apoptosis, whereas single-gene overexpression had no effect (Fig. 4H–K), underscoring the requirement for intact integrin α3β1. In vivo (Fig. 4L), orthotopic PDAC models demonstrated that ITGA3 knockdown significantly reduced metastatic dissemination, as evidenced by diminished bioluminescent signals in the abdomen and lungs (Fig. 4M), fewer and smaller metastatic nodules in the liver and lungs (Fig. 4N), and decreased CTC counts and cluster formation in peripheral blood (Fig. 4O). Quantitative analyses confirmed a marked reduction in metastatic burden across multiple organs (Fig. 4P). Together, these findings establish that ITGA3, via the integrin α3β1 complex, promotes homotypic CTC cluster formation, enhances resistance to apoptosis, and drives metastatic colonization in PDAC.

### The LAM332–ITGA3 axis regulates PDAC cell proliferation and migration

**Fig. 5:**
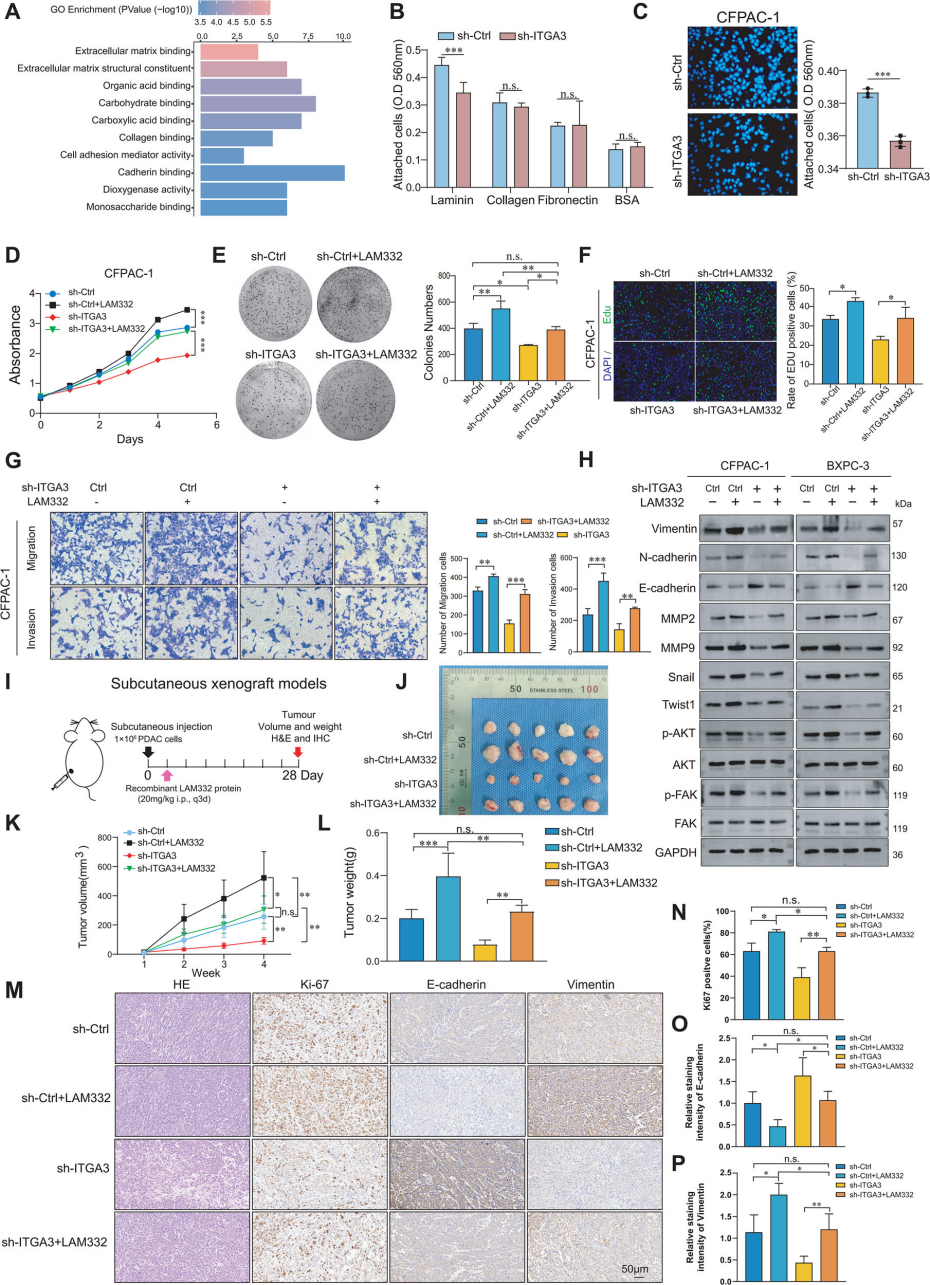
The LAM332–ITGA3 axis regulates PDAC cell proliferation and migration. **A** Functional enrichment analysis. **B** The ability of cells to bind various ECM proteins. **C** The ability of cells to bind LAM332. **D**–**F** CCK-8, colony formation, and EdU assays were conducted on PDAC cells (*n* = 3 independent experiments). **G** Transwell assays were used to assess the migration and invasion capacity of PDAC cells (*n* = 3 independent experiments). **H** Western blot analysis was performed to evaluate the expression levels of EMT-related proteins, MMPs, p-AKT, and p-FAK in PDAC cells. **I** Schematic diagram of the subcutaneous xenograft models. **J**–**L** Comparison of subcutaneous tumor volume and weight among the different groups (*n* = 5 mice/group). **M**–**P** H&E and IHC staining of Ki67, E-cadherin, and vimentin (*n* = 5 mice/group).

To elucidate the mechanisms by which ITGA3 promotes PDAC progression, we first performed GO enrichment analysis of 100 molecules associated with CTCs formation. The enriched pathways included extracellular matrix binding, Extracellular matrix structural constituent, and collagen binding, implicating ECM components as potential mediators of ITGA3-driven malignancy (Fig. 5A). Because integrins function as ECM receptors, we next evaluated candidate ECM ligands of ITGA3. Cell adhesion assays revealed that ITGA3 knockdown markedly reduced adhesion to laminin, but not to collagen or fibronectin (Fig. 5B). Given that integrin α3β1 is the canonical receptor for laminin-332 (LAM332), we examined its clinical relevance. Both mRNA and protein expression of LAMA3, LAMB3, and LAMC2 were identified as prognostic risk factors (Supplementary Fig. 3D, E), and serum LAM332 levels were significantly elevated in PDAC patients—particularly those with advanced-stage disease—compared with healthy controls (Supplementary Fig. 3F). Consistent with this, ITGA3 knockdown significantly impaired cell adhesion to LAM332 (Fig. 5C; Supplementary Fig. 3G).

To validate the functional role of the LAM332–ITGA3 axis, we treated PDAC cells with recombinant LAM332. In vitro, ITGA3 knockdown markedly suppressed pancreatic cancer cell proliferation, migration, invasion, homotypic clustering, and resistance to apoptosis, and effectively abolished the pro-tumorigenic effects induced by recombinant LAM332 (Fig. 5D–G; Supplementary Fig. 3H–M). In contrast, ITGB1 knockdown alone did not significantly inhibit the LAM332-driven enhancement of these malignant phenotypes (Supplementary Fig. 4A–E). Importantly, when treatment with an anti-integrin α3β1 antibody, LAM332 stimulation failed to promote cell proliferation, migration, invasion, homotypic clustering, or apoptosis resistance. Notably, the extent of suppression observed with

anti-integrin α3β1 antibody treatment was not significantly different from that seen with ITGA3 knockdown alone, indicating that ITGA3 is the dominant functional subunit required for LAM332 signaling, most likely acting as part of the integrin α3β1 complex (Supplementary Fig. 4A–E). These results indicate that LAM332 exerts its pro-tumorigenic effects predominantly through ITGA3, likely as part of the integrin α3β1 complex, thereby serving as a key functional mediator regulating PDAC cell proliferation, migration, invasion, clustering, and survival.

Mechanistically, LAM332 upregulated mesenchymal markers (Vimentin, N-cadherin), EMT transcription factors (Snail, Twist1), and MMP2/MMP9, while increasing phosphorylation of AKT and FAK and reducing E-cadherin. Importantly, these molecular changes were restored by LAM332 in ITGA3-deficient cells (Fig. 5H). These findings suggest that the LAM332–ITGA3 axis promotes PDAC progression through activation of the FAK/AKT signaling pathway. To further validate this mechanism, we found that pharmacological inhibition of FAK or AKT significantly attenuated PDAC cell proliferation, migration, and invasion, and largely reversed the functional effects driven by the LAM332–ITGA3 axis in vitro (Supplementary Fig. 5A–D). In vivo, subcutaneous xenograft models confirmed that LAM332 supplementation accelerated tumor growth, elevated Ki67 and vimentin expression, and decreased E-cadherin levels. Furthermore, LAM332 reversed the suppressive effects of ITGA3 knockdown on tumor proliferation and EMT (Fig. 5I–P). Importantly, pharmacological inhibition of FAK or AKT in vivo markedly suppressed tumor growth and largely reversed the LAM332–ITGA3 axis-driven effects on subcutaneous xenografts, including regulation of Ki67, Vimentin, and E-cadherin expression (Supplementary Fig. 5F–G). Collectively, these findings establish that ITGA3 exerts its oncogenic functions in PDAC primarily through interaction with LAM332, thereby driving proliferation, EMT, migration, and invasion via activation of the AKT/FAK signaling pathway.

### The LAM332–ITGA3 axis promotes homotypic CTC clustering, apoptosis resistance, and metastasis

**Fig. 6:**
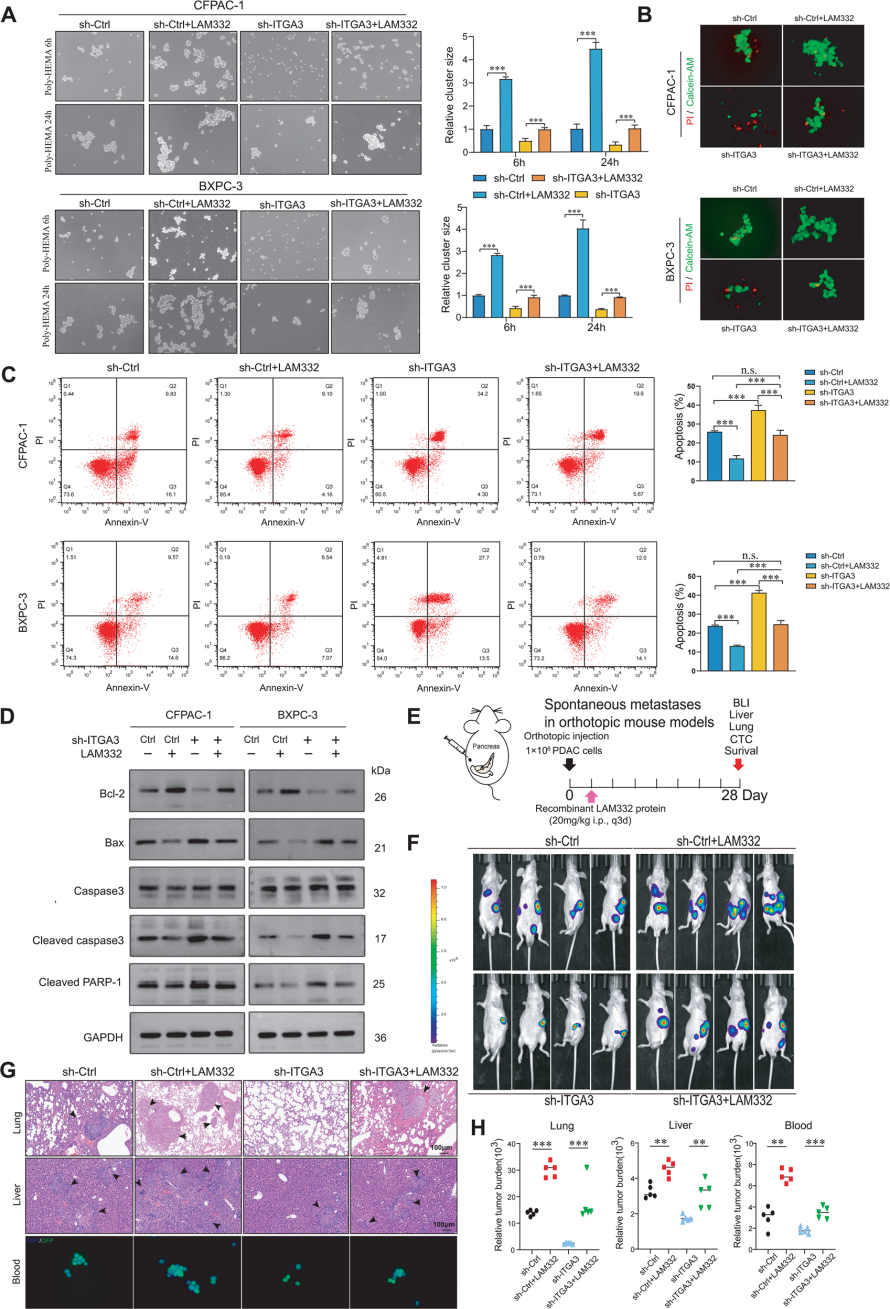
The LAM332–ITGA3 axis promotes homotypic CTC clustering, apoptosis resistance, and metastasis. **A** The cell clustering capacity of PDAC cells was determined (*n* = 3 independent experiments). **B**, **C** After 24 h of suspension culture, live/dead viability and apoptosis assays were performed for PDAC cells (*n* = 3 independent experiments). **D** The expression levels of apoptosis-related proteins were assessed via western blotting (*n* = 3 independent experiments). **E** Schematic representation of spontaneous metastases in orthotopic mouse models. **F** Representative BLI images of mice 28 days after orthotopic injection of the indicated cells (*n* = 4 mice/group). **G** Representative H&E staining and fluorescence images of the lungs and livers, and GFP-positive CTCs in the four groups of mice. **H** Metastatic burden in the liver, lung, and blood tissues of the four groups of mice at the endpoint (*n* = 5 mice/group).

Since only a small subset of CTCs survives in circulation, and homotypic CTC clusters confer a survival advantage, we next investigated whether the LAM332–ITGA3 axis contributes to these processes. In suspension culture, recombinant LAM332 significantly promoted cluster formation of PDAC cells and rescued the impaired clustering induced by ITGA3 knockdown (Fig. 6A). Live/dead staining and apoptosis assays further demonstrated that LAM332 enhanced the viability of suspended PDAC cells and restored the apoptosis resistance lost upon ITGA3 knockdown (Fig. 6B, C). Consistently, western blot analysis showed that LAM332 upregulated Bcl-2 while suppressing Bax, cleaved caspase-3, and cleaved PARP-1, thereby facilitating survival under detachment stress (Fig. 6D). In orthotopic pancreatic cancer models (Fig. 6E), LAM332 supplementation markedly increased liver and lung metastases and reversed the reduction in metastatic spread observed in ITGA3-deficient tumors, as confirmed by bioluminescence imaging and histological analysis (Fig. 6F–H). CTCs enrichment from peripheral blood revealed that LAM332 not only elevated the total number of CTCs but also increased the fraction organized into clusters, counteracting the effects of ITGA3 knockdown (Fig. 6G, H). Quantification of metastatic burden further confirmed that LAM332 restored hepatic and pulmonary colonization diminished by ITGA3 knockdown (Fig. 6G, H). Interestingly, LAM332 supplementation restored the impaired homotypic clustering ability and reduced apoptosis resistance of PDAC cells caused by ITGA3 knockdown (Supplementary Fig. 5C–E). In contrast, pharmacological inhibition of FAK or AKT significantly suppressed homotypic clustering and apoptosis resistance under suspension conditions compared with the sh-ITGA3 + LAM332 group, indicating that the LAM332–ITGA3–FAK/AKT axis regulates PDAC cell clustering and confers resistance to apoptosis (Supplementary Fig. 4C–E). Furthermore, in vivo experiments demonstrated that this axis plays a critical role in regulating CTCs formation as well as liver and lung metastasis (Supplementary Fig. 5H, I). Together, these findings demonstrate that the LAM332–ITGA3-FAK/AKT axis facilitates PDAC progression by promoting homotypic CTC clustering and enhancing apoptosis resistance in suspension, thereby supporting CTCs' survival and metastatic dissemination to distant organs.

### CAFs-derived LAM332 enhances PDAC progression via ITGA3 on tumor cells

**Fig. 7:**
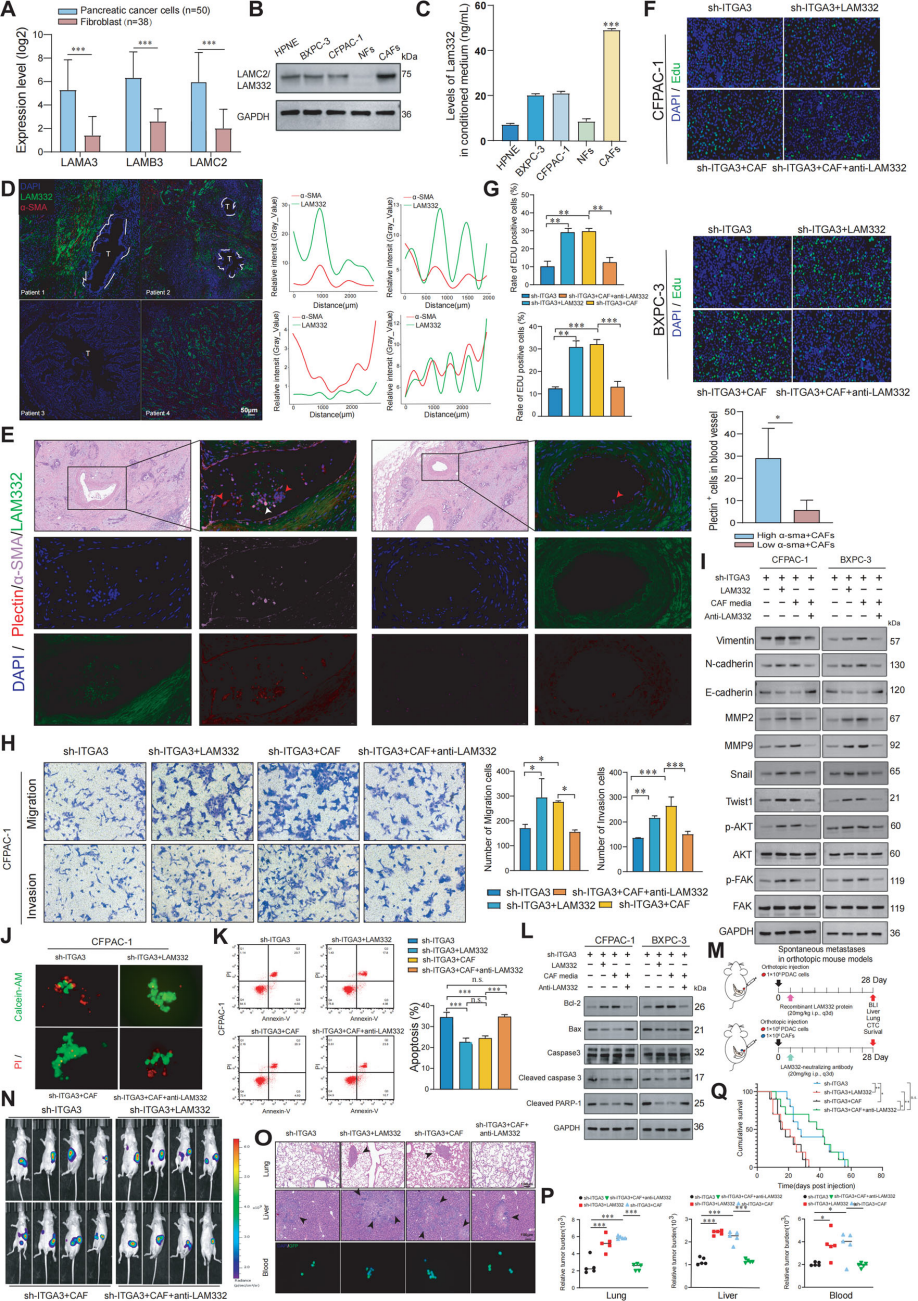
CAFs-derived LAM332 enhances PDAC progression via ITGA3 on tumor cells. **A** The expression levels of ITGA3 in human pancreatic cancer cells and fibroblast lines were analyzed on the basis of RNA-seq data from the CCLE database. **B** The expression levels of the LAM332 protein were assessed via western blotting (*n* = 3 independent experiments). **C** Secreted LAM332 was detected via ELISA (*n* = 3 independent experiments). **D** Multiplex immunofluorescence images demonstrated the origin of LAM332 from CAFs. **E** Multiplex immunofluorescence images demonstrated the association between CAFs and CTCs in the peritumoral vasculature. **F,** **G** PDAC cells were harvested for the EdU assay (*n* = 3 independent experiments). **H** The cell migration and invasion capacities of PDAC cells were determined via transwell assays (*n* = 3 independent experiments). **I** The expression levels of EMT-related proteins, MMPs, p-AKT, and p-FAK were assessed via western blotting (*n* = 3 independent experiments). **J**, **K** After 24 h of suspension culture, live/dead viability and apoptosis assays were performed for PDAC cells (*n* = 3 independent experiments). **L** The expression levels of apoptosis-related proteins were assessed via western blotting (*n* = 3 independent experiments). **M** Schematic representation of spontaneous metastases in orthotopic mouse models. **N** Representative images of bioluminescence imaging (BLI) of mice at 28 days after orthotopic injection of the indicated cells (*n* = 4 mice/group). **O** Representative H&E staining/fluorescence images of the lungs and livers/GFP-positive CTCs of the four groups of mice. **P** Metastatic burden in the liver, lungs, and blood of the four groups of mice at the endpoint (*n* = 5 mice/group). **Q** Kaplan‒Meier survival analysis was performed to evaluate the overall survival of the mice (*n* = 10 mice/group).

Given that the LAM332–ITGA3 axis promotes CTC clustering and metastasis, we next investigated the cellular origin of LAM332. CCLE analysis showed that LAMA3, LAMB3, and LAMC2 were expressed at higher levels in PDAC cells than in fibroblasts (Fig. 7A). However, western blotting and ELISA revealed markedly higher levels of LAM332 in CAFs compared with PDAC cells, HPNE cells, or NFs (Fig. 7B, C). Immunofluorescence staining of PDAC patient samples further confirmed that LAM332 was enriched in α-SMA⁺ CAFs, suggesting that CAFs are the major source of LAM332 in the tumor microenvironment (Fig. 7D). Importantly, patients with higher numbers of α-SMA⁺ CAFs exhibited increased vascular LAM332 levels and more CTCs, implicating CAFs-derived LAM332 in promoting CTCs abundance and survival (Fig. 7E). Functionally, recombinant LAM332 or CAF-conditioned medium (CAF-CM) restored proliferation, migration, and invasion in ITGA3-knockdown PDAC cells, whereas neutralization of LAM332 abrogated these effects (Fig. 7F–H; Supplementary Fig. 6A–F). Both CAF-CM and recombinant LAM332 induced EMT and activated AKT/FAK signaling, as evidenced by upregulation of vimentin, N-cadherin, Snail, Twist1, MMP2, and MMP9, phosphorylation of AKT and FAK, and downregulation of E-cadherin. These effects were reversed by LAM332 blockade (Fig. 7I). In suspension cultures, CAF-CM or recombinant LAM332 restored clustering and apoptosis resistance in ITGA3-knockdown cells, while neutralization of LAM332 impaired clustering and promoted apoptosis (Fig. 7J, K; Supplementary Fig. 6,G–J). Consistently, CAF-CM or recombinant LAM332 increased Bcl-2 and reduced Bax, cleaved caspase-3, and cleaved PARP-1, effects abolished by LAM332 inhibition (Fig. 7L). In vivo, recombinant LAM332 or CAFs accelerated subcutaneous tumor growth, accompanied by higher Ki67 and vimentin, and reduced E-cadherin, whereas LAM332 blockade mitigated these effects (Supplementary Fig. 6K–O). Similarly, in orthotopic pancreatic tumor models, CAF-CM or recombinant LAM332 enhanced CTC cluster formation, CTC survival, and liver/lung metastasis, whereas LAM332 neutralization reduced CTC numbers and metastatic burden, and prolonged survival (Fig. 7M–Q). Collectively, these results establish CAFs as the dominant source of LAM332 in PDAC and demonstrate that CAFs-derived LAM332 promotes CTCs formation, EMT, survival, and metastasis via ITGA3. Blocking LAM332 effectively suppresses these malignant processes and improves survival, underscoring CAFs-derived LAM332 as a promising therapeutic target.

## Discussion

Metastasis is the primary cause of mortality in PDAC patients [24]. The formation and survival of CTCs in the bloodstream are critical factors for metastasis of PDAC; however, the underlying mechanisms remain poorly understood [25]. Our study revealed that CAFs-derived LAM332 facilitates the formation of CTCs and homotypic CTC clustering through ITGA3 and modulates the expression of apoptosis-associated proteins, ultimately promoting the survival of CTCs and fostering liver and lung metastasis in PDAC.

By analyzing proteomic data from 20 FFPE samples combined with RNA-seq datasets associated with CTCs and prognosis in PDAC, ITGA3 was identified as a potential driver of CTC formation and survival in PDAC. Growing evidence indicates that ITGA3 is closely associated with the malignant progression of pancreatic cancer [16, 26]. We observed notably high expression of ITGA3 in the TIF and detected a significant positive correlation between ITGA3 expression and the number of CTCs in the vasculature of PDAC tissues. Our results also demonstrate that the expression of ITGA3 in PDAC cells is associated with the formation and survival of CTCs in vivo and in vitro. These findings suggest that ITGA3 may contribute to the malignant progression of PDAC and poor prognosis by promoting the formation of CTCs.

How does ITGA3 promote CTC formation in PDAC? In our study, clinical tissue samples and in vitro experiments demonstrated that ITGA3 promotes PDAC cell proliferation, drives EMT, and enhances the expression of MMP2 and MMP9, thereby facilitating tumor cell detachment from the primary site. Aberrant reactivation of EMT is associated with the malignant characteristics of tumor cells during cancer progression and metastasis, promoting cell detachment from the primary tumor to form CTCs [27]. Consistently, previous studies have demonstrated that FOXD1-mediated EMT promotes breast cancer cell detachment from the primary tumor to form CTCs [8]. Furthermore, CTCs are highly susceptible to apoptosis upon entering the circulation. Existing evidence suggests that CTC clusters exhibit significantly enhanced survival and metastatic potential compared to single CTC, with metastatic efficiency 20–100 times greater [23]. Intriguingly, our findings revealed that the cell surface protein ITGA3 contributes to the formation of homotypic CTC clusters and modulates the expression of apoptosis-related proteins, thereby promoting CTCs' survival and facilitating distant metastasis in PDAC. These insights advance the understanding of mechanisms driving homotypic CTC cluster formation. It is well-established that the dense fibrotic stroma characteristic of PDAC creates a tumor microenvironment (TME) that plays a crucial role in tumor initiation and progression through reciprocal interactions between tumor cells and the TME [28]. CAFs, the most abundant stromal cells within the TME, secrete extracellular matrix (ECM) proteins essential for TME remodeling, thereby promoting tumor proliferation, migration, and invasion in various cancers [29]. Integrins, as key ECM receptors, facilitate bidirectional transmembrane signaling and mediate communication between tumor cells and CAFs [30]. Here, we identified that CAFs at the primary tumor site deliver oncogenic signals through LAM332 via ITGA3, activating downstream FAK/AKT signaling pathways in PDAC cells. Moreover, prior studies have shown that CAFs can migrate from the primary tumor into the peripheral circulation, where they contribute to CTC clustering and enhance tumor cell survival, facilitating metastasis [31]. Consistently, our clinical multiparametric immunofluorescence analysis of PDAC tissues revealed that CAFs within the vasculature secrete LAM332, which positively correlates with CTC abundance. Under suspension culture conditions, we demonstrated that neutralizing LAM332 in CAFs-conditioned media suppressed PDAC cell clustering via ITGA3, induced apoptosis, reduced metastatic burden in the liver and lungs, and improved survival in nude mice bearing the tumor. Collectively, these results underscore the pivotal role of CAFs-derived LAM332 in promoting homotypic CTC cluster formation and survival through ITGA3, thereby driving distant metastasis in PDAC.

Currently, various strategies have attempted to disrupt tumor–stroma interactions by targeting integrins or ECM components, thereby inhibiting tumor invasion and metastasis [32]. For instance, Cilengitide, an αvβ3/αvβ5 integrin inhibitor, suppresses tumor progression by blocking integrin–ECM binding, while the α5β1-targeting monoclonal antibody MINT1526A has been evaluated in early-phase clinical trials for its ability to interfere with integrin–ECM interactions [33, 34]. In comparison, our study identifies the CAFs‑derived LAM332–ITGA3 axis as a more mechanism-driven and tumor-specific therapeutic target. Selective inhibition of LAM332 secretion by CAFs or blockade of ITGA3 function effectively impairs CTCs aggregation and survival, providing a novel strategy for the prevention and treatment of PDAC metastasis. Moreover, these findings suggest that targeting the CAFs‑LAM332–ITGA3 axis could serve as a valuable complement or optimization to existing anti-stroma or anti-integrin therapies. Monitoring or selectively inhibiting this pathway may offer promising strategies for assessing metastatic risk and treating PDAC. Nevertheless, this study does not address the role of heterotypic CTC clusters in CTC survival, nor does it elucidate the precise mechanisms underlying the interaction between PDAC cells and CAFs, warranting further investigation.

## Conclusion

In conclusion, this study elucidates novel mechanisms underlying CTCs formation and survival, offering insights into the therapeutic potential of targeting PDAC. Targeting CAFs-derived LAM332, which interacts with ITGA3 on the surface of PDAC cells, could be a promising approach for assessing metastatic risk, predicting prognosis, and preventing metastasis in patients with PDAC.

## Supplementary information

Supplementary figures and tables

Original Western blots.

## Data Availability

The analyzed datasets generated during the study are available from the corresponding authors on reasonable request.
