# Supplementary material for: CAFs-derived LAM332 promotes CTCs formation and survival via ITGA3 and contributes to the metastasis of pancreatic ductal adenocarcinoma
Source: Cell Death Dis. 2026 Mar 25;17(1):369. doi: 10.1038/s41419-026-08642-z (PMC13039154; doi:10.1038/s41419-026-08642-z)

**CAFs-Derived LAM332 Promotes CTCs Formation and Survival via ITGA3 and Contributes to the Metastasis of Pancreatic Ductal Adenocarcinoma**

Supplementary information

Supplementary Tables S1–S6

Supplementary Figures S1–S6

Supplementary Table 1. The sequences of primers used in the study.

ITGA3: 5’-CGCTGTCTTCCACGGCTTCTTC

3’-CGGAGTTTGTCACGGAGGTTGTC

ITGB1: 5’- TGGGCTTTACGGAGGAAGTAGAGG

3’- GACACTTGGGACTTTCAGGGATGC

GAPDH: 5’-TGACATCAAGAAGGTGGTGAAGCAG

3’-GTGTCGCTGTTGAAGTCAGAGGAG

| Supplementary Table 2. **Information on antibodies used in the study.** | | | | | | |
| --- | --- | --- | --- | --- | --- | --- |
| Antibodies | Source | Host | WB(dilution ratio) | IHC(dilution ratio) | | IF(dilution ratio) |
| anti-ITGA3 | Abcam,ab8988 | Mouse | 1/3500 | 1/600 | 1/600 | |
| anti-ITGB1 | Abcam,ab52971 | Rabbit | 1/10000 | NA | NA | |
| anti-Vimentin | Abcam,ab92547 | Rabbit | 1/3000 | 1/500 | 1/300 | |
| anti-N-cadherin | Abcam,ab76011 | Rabbit | 1/10000 | 1/500 | NA | |
| anti-E-cadherin | Abcam,ab40772 | Rabbit | 1/30000 | 1/500 | NA | |
| anti-Snail | Abcam,ab85936 | Rabbit | 1/5000 | NA | NA | |
| anti-Twist1 | Abcam,ab175430 | Mouse | 1/2000 | NA | NA | |
| anti-MMP2 | Abcam,ab92536 | Rabbit | 1/5000 | NA | NA | |
| anti-MMP9 | Abcam,ab76003 | Rabbit | 1/20000 | NA | NA | |
| anti-AKT | Abcam,ab8805 | Rabbit | 1/500 | NA | NA | |
| anti-P-AKT | Abcam,ab81283 | Rabbit | 1/10000 | NA | NA | |
| anti-FAK | Abcam,ab40794 | Rabbit | 1/2000 | NA | NA | |
| anti-p-FAK | Abcam,ab81298 | Rabbit | 1/1000 | NA | NA | |
| anti-Bax | Abcam,ab32503 | Rabbit | 1/10000 | NA | NA | |
| anti-BCL-2 | Abcam,ab182858 | Rabbit | 1/2000 | NA | NA | |
| anti-caspace-3 | Abcam,ab32351 | Rabbit | 1/5000 | NA | NA | |
| anti-cleaved-caspace-3 | Abcam,ab32042 | Rabbit | 1/500 | NA | NA | |
| anti-cleaved PARP-1 | Abcam,ab32064 | Rabbit | 1/10000 | NA | NA | |
| anti-GAPDH | Abcam, ab8245 | Mouse | 1/10000 | NA | NA | |
| anti-Plectin | Abcam,ab312312 | Rabbit | NA | NA | 1/50 | |
| anti-LAMC2 | Abcam,ab274376 | Rabbit | 1/1000 | NA | 1/100 | |
| anti-α-SMA | Servicebio, GB13044 | Rabbit | 1/1000 | NA | 1/1000 | |

Supplementary Table 3. **The sequences of siRNAs used in the study.**

ITGA3-Homo-3511#1: 5’- CCUGGUGUGACUUCUUUAATT

3’-UUAAAGAAGUCACACCAGGTT

ITGA3-Homo-3135#2: 5’- GCACCUUCAUCGAGGAUUATT

3’-UAAUCCUCGAUGAAGGUGCTT

ITGA3-Homo-1468#3: 5’- CCAGGAUGGAUUUCAGGAUTT

3’-AUCCUGAAAUCCAUCCUGGT

Supplementary Table 4. DEPs Between CTC and No CTC Groups.

| Gene Name | CTC/no-CTC | P value |
| --- | --- | --- |
| SERPINB5 | 3.097093508 | 0.018834505 |
| CLEC11A | 2.952533857 | 0.020675878 |
| LAMA3 | 2.874453169 | 0.03910666 |
| PLOD1 | 2.439573243 | 0.002018929 |
| HK3 | 2.420784596 | 0.019110641 |
| PPP1R13L | 2.405711118 | 7.61171E-05 |
| SPTBN4 | 2.340892274 | 0.008349623 |
| PAIP1 | 2.31326543 | 0.030704168 |
| GSDME | 2.313164822 | 0.029149555 |
| HTRA1 | 2.271943487 | 0.034754658 |
| ITGA2 | 2.262060392 | 0.000256206 |
| FAP | 2.246713455 | 0.017592543 |
| FHL3 | 2.192136177 | 0.002070709 |
| PPP4R1 | 2.176084284 | 0.003621133 |
| NNMT | 2.100165717 | 0.002961821 |
| PTK7 | 2.023211564 | 0.012330325 |
| B4GALT1 | 1.991032881 | 0.006291013 |
| ALOX5AP | 1.977147187 | 0.003052878 |
| NBEAL2 | 1.963780917 | 0.026631586 |
| IGFBP7 | 1.943005251 | 0.010759882 |
| CAPG | 1.939214965 | 0.011644615 |
| ITGA3 | 1.897175731 | 0.019217949 |
| COL12A1 | 1.876524098 | 0.016899208 |
| LAD1 | 1.832620078 | 0.005111178 |
| ITPR3 | 1.817523449 | 0.006677493 |
| S100A11 | 1.811613353 | 0.001596482 |
| VILL | 1.784961992 | 0.049317057 |
| P4HA1 | 1.782182098 | 0.045723662 |
| PRKCD | 1.774358914 | 0.019817405 |
| CRTAP | 1.758995228 | 0.002448969 |
| SP100 | 1.73938561 | 0.010830387 |
| RAB11FIP1 | 1.73818885 | 0.003443732 |
| DUSP23 | 1.737947413 | 0.000459929 |
| CPOX | 1.717255553 | 0.009829819 |
| DTX3L | 1.714613271 | 0.010501143 |
| PDCD10 | 1.691503671 | 0.03302322 |
| CNN2 | 1.670811128 | 0.016161904 |
| RBM42 | 1.66464542 | 0.033269926 |
| TNFAIP2 | 1.658210366 | 0.031774301 |
| MYOF | 1.645004323 | 0.021405002 |
| PIP4K2C | 1.636940526 | 0.030957567 |
| ASPH | 1.635702533 | 0.001559512 |
| ACOX1 | 1.624358996 | 0.021368362 |
| NCEH1 | 1.617012149 | 0.019254982 |
| SERPINH1 | 1.612164234 | 0.027317567 |
| PLOD3 | 1.611255632 | 0.023497194 |
| NAMPT | 1.606095679 | 0.009513185 |
| RAB32 | 1.601452651 | 0.004695176 |
| LPCAT2 | 1.601063701 | 0.008425595 |
| FLNB | 1.591265585 | 0.000529512 |
| GTF2F1 | 1.589964441 | 0.036887422 |
| ALDH1A3 | 1.589772208 | 0.031830969 |
| HSPH1 | 1.585981287 | 0.010951067 |
| INF2 | 1.571267012 | 0.009169167 |
| PLXDC2 | 1.562736378 | 0.016316683 |
| IGF2R | 1.558168684 | 0.008544809 |
| ARHGEF40 | 1.557537663 | 0.041655441 |
| TAGLN2 | 1.554044475 | 0.003861488 |
| S100A16 | 1.55139985 | 0.043691159 |
| DOCK7 | 1.546392744 | 0.001595811 |
| GSDMD | 1.545305622 | 0.007721053 |
| ARHGAP1 | 1.531230035 | 0.001725372 |
| MCU | 1.52353101 | 0.037538624 |
| FKBP9 | 1.52073762 | 0.016498279 |
| NUP160 | 1.518789028 | 0.031925748 |
| MAPK14 | 1.512873415 | 0.007470583 |
| PDLIM5 | 1.510367075 | 0.003800825 |
| RASA1 | 1.510190628 | 0.01275174 |
| PFKP | 1.508320453 | 0.028833566 |
| ALOX5 | 1.504623515 | 0.015866451 |
| TKFC | -1.503332807 | 0.023499155 |
| ELN | -1.523687946 | 0.025097597 |
| SOD3 | -1.527860758 | 0.01107894 |
| SUCLG2 | -1.549547671 | 0.032740018 |
| KBTBD11 | -1.571556234 | 0.009723477 |
| PLVAP | -1.577385004 | 0.023007708 |
| MAOA | -1.581803587 | 0.02061062 |
| ACSF3 | -1.585177766 | 0.008943825 |
| TM9SF1 | -1.599824789 | 0.008013624 |
| RAB27A | -1.603414758 | 0.021049751 |
| TMT1A | -1.603563278 | 0.028102556 |
| FXN | -1.603649499 | 0.036820114 |
| CD34 | -1.607875477 | 0.020902842 |
| ALDH1A1 | -1.624162043 | 0.04590124 |
| ANPEP | -1.627476556 | 0.039887817 |
| OGN | -1.675738189 | 0.048037621 |
| OLFML1 | -1.702510073 | 0.046953681 |
| CLIC2 | -1.703322129 | 0.001344437 |
| ISOC2 | -1.728120278 | 0.017446755 |
| COX6A1 | -1.786285213 | 0.012480614 |
| DBT | -1.838579332 | 0.028749424 |
| TNXB | -1.853550554 | 0.026918496 |
| IGHM | -1.870854621 | 0.025238299 |
| TCEA3 | -1.89772549 | 0.032063159 |
| ECI2 | -1.943625597 | 0.046542116 |
| ALG2 | -1.964918855 | 0.016939137 |
| NUCB2 | -2.245860165 | 0.039717634 |
| CRELD2 | -2.382490284 | 0.003598267 |
| PRPH | -2.38855812 | 0.025450417 |
| ACADL | -2.620693836 | 0.048213412 |

Supplementary Table 5. **Identification of 70 prognostic genes by univariate Cox regression analyses.**

| id | HR | HR.95L | HR.95H | pvalue |
| --- | --- | --- | --- | --- |
| LAMC2 | 1.36507439 | 1.135627164 | 1.640880166 | 0.000917872 |
| LAMB3 | 1.350903364 | 1.098939889 | 1.66063669 | 0.004293543 |
| SLC2A1 | 1.41836838 | 1.130198841 | 1.780013204 | 0.00255959 |
| LAMA3 | 1.506977929 | 1.10003567 | 2.06446258 | 0.010660299 |
| CDH3 | 1.396055103 | 1.096351994 | 1.777686237 | 0.006809475 |
| ITGB4 | 1.554952072 | 1.154050302 | 2.0951218 | 0.003710458 |
| SERPINB5 | 1.254687277 | 1.041037875 | 1.51218337 | 0.01720751 |
| KRT19 | 1.309461735 | 1.051003278 | 1.631479245 | 0.016243384 |
| FBXO32 | 1.700156236 | 1.285818646 | 2.248008486 | 0.000196125 |
| ITGA2 | 1.252982012 | 1.018378511 | 1.541631041 | 0.032995498 |
| MICAL2 | 1.648134421 | 1.109180067 | 2.448968523 | 0.013406035 |
| PLEK2 | 1.345179051 | 1.01966761 | 1.77460445 | 0.035927248 |
| RUNX2 | 1.465588669 | 1.054740942 | 2.036471763 | 0.022756489 |
| SCEL | 1.210885953 | 1.020170903 | 1.437254078 | 0.028644319 |
| POSTN | 1.300656917 | 1.059379153 | 1.596886639 | 0.012040373 |
| FGD6 | 1.94055895 | 1.386543474 | 2.71594011 | 0.000110896 |
| FN1 | 1.420996267 | 1.065430172 | 1.895225463 | 0.016788213 |
| ECT2 | 1.48869795 | 1.082365199 | 2.047572842 | 0.014419611 |
| S100A16 | 1.664285199 | 1.122061291 | 2.468532908 | 0.011324079 |
| ITGA3 | 1.668401587 | 1.31648888 | 2.114384631 | 2.29E-05 |
| SULF1 | 1.378747299 | 1.017955563 | 1.86741365 | 0.037992486 |
| MET | 1.377298656 | 1.094216505 | 1.733616319 | 0.00639227 |
| PLAU | 1.553019644 | 1.100386121 | 2.191839727 | 0.012274749 |
| CDCP1 | 1.624599147 | 1.191515675 | 2.215096658 | 0.002157128 |
| OSBPL3 | 1.475057003 | 1.040325699 | 2.091453825 | 0.029118374 |
| ITGB6 | 1.265541078 | 1.056256521 | 1.51629286 | 0.010668321 |
| IL1RAP | 1.471061246 | 1.072431535 | 2.017864188 | 0.016683308 |
| ERO1A | 1.503808577 | 1.150696503 | 1.96527949 | 0.002808845 |
| HK2 | 1.520257228 | 1.142671199 | 2.022613366 | 0.004033778 |
| MMP14 | 1.81611383 | 1.277222375 | 2.58237681 | 0.000892591 |
| SYTL2 | 0.688983004 | 0.500893691 | 0.947701256 | 0.022010707 |
| ANLN | 1.595086714 | 1.277952596 | 1.990920191 | 3.65E-05 |
| ADAMTS12 | 1.448352604 | 1.063079614 | 1.973253214 | 0.018892846 |
| LMO3 | 0.648183033 | 0.47793153 | 0.8790825 | 0.005288103 |
| BTG2 | 0.721858747 | 0.541346995 | 0.96256201 | 0.026429237 |
| ARNTL2 | 1.617321163 | 1.23628253 | 2.115800943 | 0.000452583 |
| KRT17 | 1.527515796 | 1.164246907 | 2.00413202 | 0.002231795 |
| ENO2 | 1.374977625 | 1.023951186 | 1.846341403 | 0.034230831 |
| CENPF | 1.515218993 | 1.168492083 | 1.964830253 | 0.00172157 |
| LOXL2 | 1.500106 | 1.129442364 | 1.99241509 | 0.005101197 |
| PROX1 | 0.722328857 | 0.524419211 | 0.994927278 | 0.046470498 |
| DCBLD2 | 1.801167043 | 1.39382529 | 2.327553345 | 6.85E-06 |
| MELK | 1.470963305 | 1.119790069 | 1.932266686 | 0.005555726 |
| TPX2 | 1.914269102 | 1.4422195 | 2.540824191 | 6.97E-06 |
| TOP2A | 1.51659429 | 1.180950967 | 1.947632292 | 0.001101922 |
| ANXA8 | 1.331771385 | 1.110959633 | 1.596471167 | 0.001951004 |
| MUC16 | 1.26166176 | 1.065409084 | 1.494064974 | 0.007050383 |
| MKI67 | 1.677167428 | 1.257275744 | 2.237290105 | 0.000436143 |
| GREM1 | 1.437585941 | 1.019860433 | 2.026407998 | 0.038243082 |
| IGF2BP3 | 1.560226793 | 1.238223794 | 1.965967425 | 0.000162104 |
| TGM2 | 1.58184195 | 1.128111911 | 2.218063588 | 0.007840103 |
| CXCL5 | 1.310712598 | 1.080437526 | 1.590066499 | 0.006054724 |
| FLRT2 | 0.654684324 | 0.4864402 | 0.881118714 | 0.005188873 |
| ASPM | 1.424410108 | 1.092675963 | 1.856858049 | 0.00891855 |
| DLGAP5 | 1.84426358 | 1.32456983 | 2.567858695 | 0.000289612 |
| KRT7 | 1.535409786 | 1.207994568 | 1.951567724 | 0.000458017 |
| DKK1 | 1.448964432 | 1.14504708 | 1.833547249 | 0.002017237 |
| RGN | 0.73698777 | 0.545194454 | 0.996251831 | 0.04721335 |
| NT5E | 1.414407283 | 1.137533126 | 1.758672268 | 0.001812667 |
| TGFBI | 1.611934554 | 1.191460621 | 2.180796377 | 0.001962078 |
| GPX2 | 0.785506556 | 0.624615129 | 0.987841184 | 0.03896291 |
| KRT6A | 1.251017038 | 1.085754957 | 1.441433558 | 0.001947446 |
| MUC13 | 0.804729606 | 0.677783523 | 0.95545217 | 0.01313014 |
| SRPX2 | 1.528150238 | 1.085339618 | 2.15162435 | 0.015138153 |
| PXDN | 1.416630372 | 1.048236217 | 1.914493678 | 0.023418399 |
| MUC17 | 0.768401653 | 0.653775001 | 0.903125845 | 0.001392677 |
| ADH1B | 0.77802122 | 0.653325339 | 0.926516978 | 0.004857457 |
| TSPAN8 | 0.792823528 | 0.668168368 | 0.940734666 | 0.0078149 |
| DSG3 | 1.295944986 | 1.110484879 | 1.512378457 | 0.001002276 |
| C7 | 0.711701975 | 0.611803488 | 0.82791241 | 1.05E-05 |

Supplementary Table 6. **Univariate Cox regression analyses of risk factors for OS and RFS.**

|  | OS | | RFS | |
| --- | --- | --- | --- | --- |
| Variable | HR(95% CI) | P Value | HR(95% CI) | P Value |
| Gender |  |  |  |  |
| Female |  |  |  |  |
| Male | 0.74(0.41-1.37) | 0.340 | 0.76(0.44-1.33) | 0.338 |
| Age |  |  |  |  |
| >65 |  |  |  |  |
| ≥65 | 1.05(0.58-1.91) | 0.880 | 1.07(0.62-1.85) | 0.811 |
| Tumor site |  |  |  |  |
| body/tail |  |  |  |  |
| Head | 0.70(0.36-1.35) | 0.282 | 0.78(0.43-1.43) | 0.430 |
| Differentiated degree |  |  |  |  |
| I+II |  |  |  |  |
| III | 1.91(0.93-3.92) | 0.079 | 2.30(1.17-4.51) | 0.015 |
| Tumor size（cm） |  |  |  |  |
| <3 |  |  |  |  |
| ≥3 | 1.65(0.86-3.18) | 0.135 | 1.81(1.00-3.28) | 0.049 |
| Adjacent organs invasion |  |  |  |  |
| Negative |  |  |  |  |
| Positive | 1.86(0.93-3.70) | 0.077 | 1.50(0.83-2.71) | 0.177 |
| Lymph node metastasis or vascular invasion |  |  |  |  |
| Negative |  |  |  |  |
| Positive | 1.64(0.88-3.06) | 0.120 | 1.85(1.05-3.26) | 0.034 |
| ITGA3 expression |  |  |  |  |
| Low |  |  |  |  |
| High | 2.21(1.20-4.07) | 0.011 | 3.25(1.84-5.76) | 0.000 |

# **Supplementary Figure 1. Batch effect assessment and single-cell analysis of ITGA3 expression in pancreatic cancer datasets.** (A) PCA analysis of GSE28735 and GSE62452 data before and after normalisation. (B) Umap plot of cell clustering in the PAAD_CRA001160 dataset. (C)Violin plot of ITGA3 expression in different cell subpopulations in the PAAD_CRA001160 dataset.(D) Umap plot of cell clustering in the GSE154778 dataset.(E) Umap plot of ITGA3 expression in the GSE154778 dataset.

#
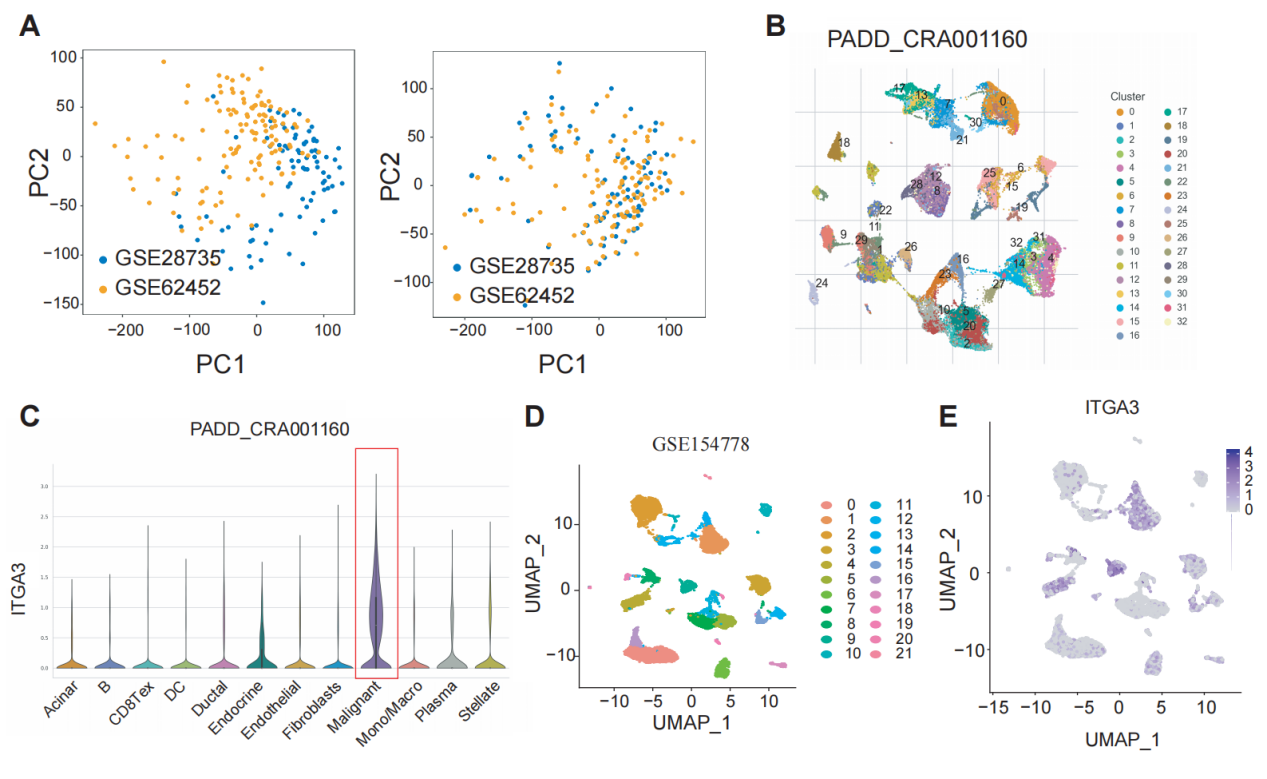


# **Supplementary Figure 2. Validation of ITGA3 genetic manipulation and functional characterization in pancreatic cancer cells.** (A) Examination of ITGA3 expression levels in HPNE cells and pancreatic cancer cells through western blotting and qRT‒PCR (n = 3 independent experiments). (B)(C)Validation of the ability of 3 siRNAs to knock down ITGA3 (n = 3 independent experiments). (D) (E)Stable knockdown of ITGA3 via lentivirus-mediated shRNA technology (n = 3 independent experiments). (F) BXPC-3 cells were harvested for the EdU assay (n = 3 independent experiments). (G) (H)Stable overexpression of ITGA3 and ITGB1 in CAPAN-2 cells (n = 3 independent experiments). (I)(J) Cell migration and invasion capacity were determined via a wound healing assay and a transwell assay (n = 3 independent experiments).


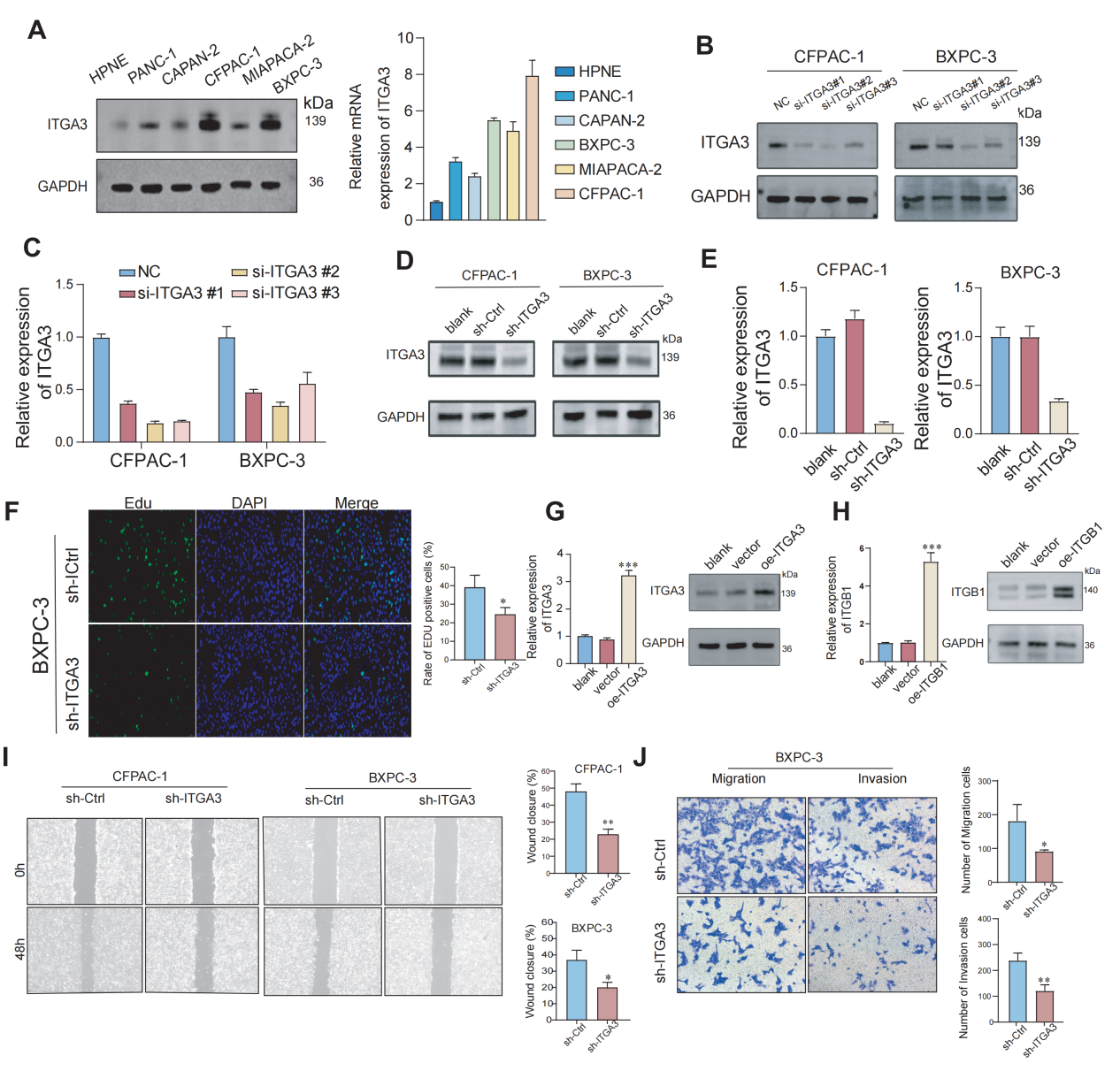


**Supplementary Figure 3**. **The LAM332–ITGA3 axis promotes pancreatic cancer cell malignant phenotypes.** (A) Cell clustering capacity was assessed in ITGA3-knockdown and control cells (n = 3 independent experiments). (B)(C) Live/dead viability assays and apoptosis assays were performed for cells after 24 hours of suspension culture (n = 3 independent experiments). (D) Forest plot demonstrating that LAMA3, LAMB3 and LAMC2 (mRNA expression levels) are risk factors affecting the prognosis of pancreatic cancer patients (GSE28735 and GSE62452) (n=114). (E) Forest plot showing that LAMA3, LAMB3 and LAMC2 (protein expression levels) are risk factors affecting the prognosis of pancreatic cancer (CPTAC database PDC000248) (n=140). (F)Comparison of serum protein levels of LAM332 between healthy controls (n=20), localized PDAC patients (n=14) and advanced PDAC patients (n=6). (G) Ability of cells to bind LAM332. (H)(I)(J) CCK-8, colony formation, and EdU assays were conducted with PDAC cells (n = 3 independent experiments). (K)(L)(M) Cell migration and invasion capacity were determined via a wound healing assay and a transwell assay (n = 3 independent experiments).


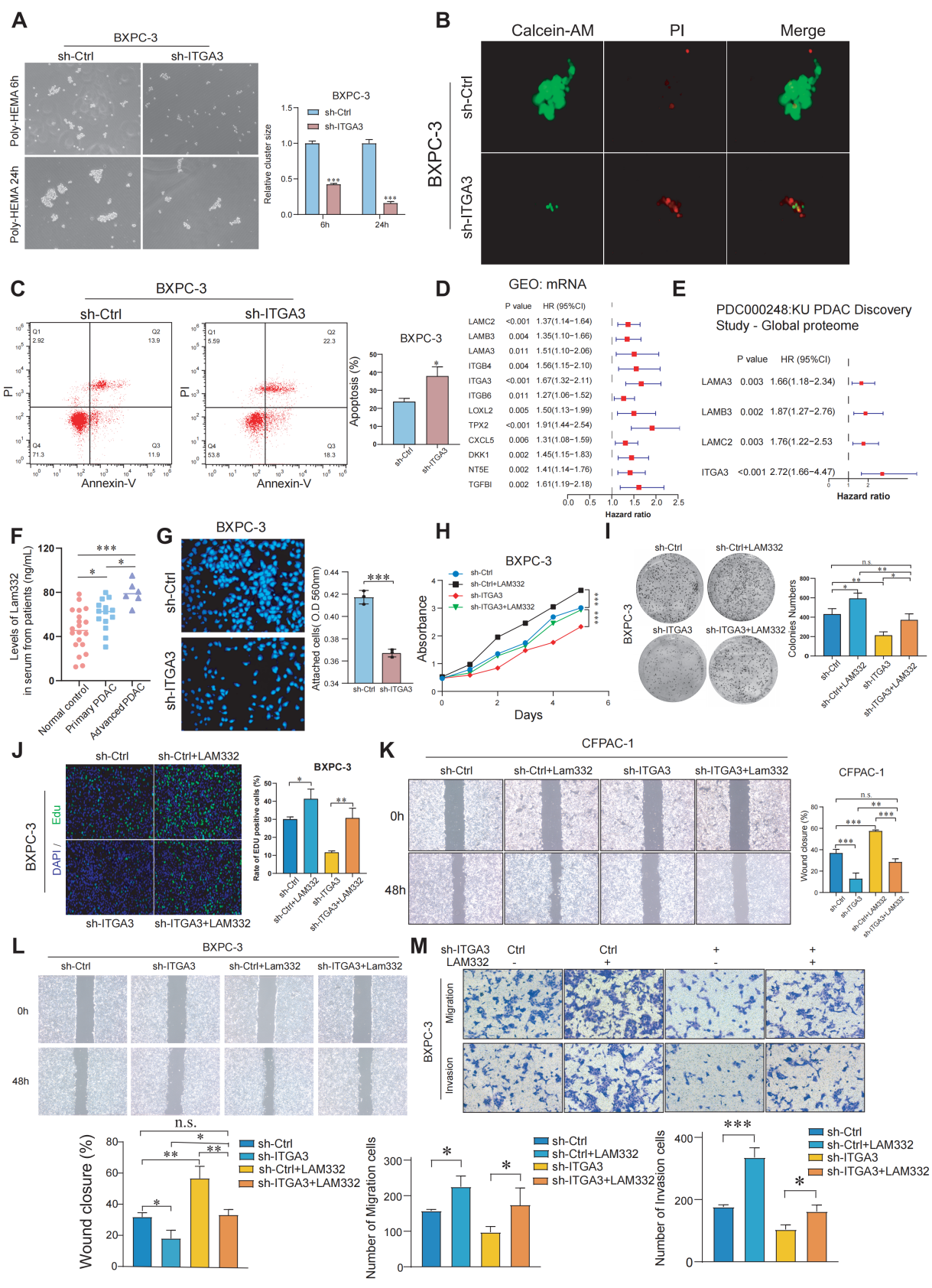


**Supplementary Figure 4. LAM332 promotes malignant phenotypes mainly via ITGA3, likely within the integrin α3β1 complex.** (A)(B) PDAC cells of four groups (LAM332+sh-ctrl, LAM332+sh-ITGA3, LAM332+sh-ITGB1, and LAM332+anti-integrin α3β1 abtibody) were harvested for the CCK-8 cell proliferation assay and EdU assay (n = 3 independent experiments). (C)(D)(E) PDAC cells of four groups (LAM332+sh-ctrl, LAM332+sh-ITGA3, LAM332+sh-ITGB1, and LAM332+anti-integrin α3β1 abtibody) were harvested for the transwell migration/invasion assay and cell clustering assay (n = 3 independent experiments).


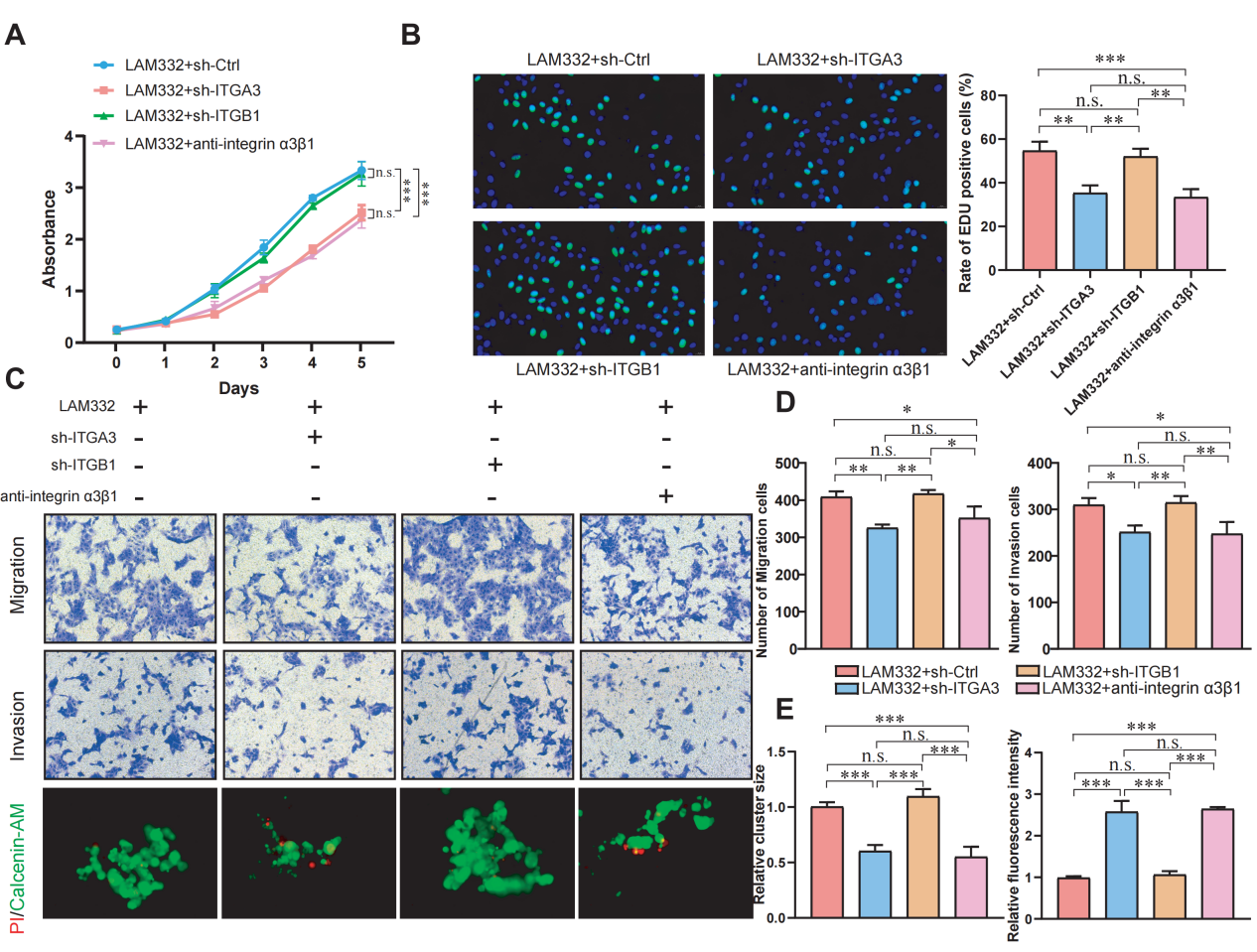


**Supplementary Figure 5. ITGA3 drives LAM332-induced malignant phenotypes through the AKT/FAK signaling pathway.** (A)(B) PDAC cells of four groups (sh-ITGA3, sh-ITGA3+LAM332, sh-ITGA3+LAM332+FAK inhibitor and sh-ITGA3+LAM332+AKT inhibitor) were harvested for the CCK-8 cell proliferation assay and EdU assay (n = 3 independent experiments). (C)(D)(E) PDAC cells of four groups (LAM332+sh-ctrl, LAM332+sh-ITGA3, LAM332+sh-ITGB1, and LAM332+sh-ITGA3&ITGB1) were harvested for the transwell migration/invasion assay and cell clustering assay (n = 3 independent experiments). (F) Comparison of subcutaneous tumor volume and weight among the different groups (n= 5 mice/group). (G)IHC staining of Ki67, E-cadherin, and Vimentin (n= 5 mice/group). (H)Schematic representation of spontaneous metastases in orthotopic mouse models. (I)Metastatic burden in the liver, lung, and blood tissues of the four groups of mice at the endpoint (n= 5 mice/group).

#
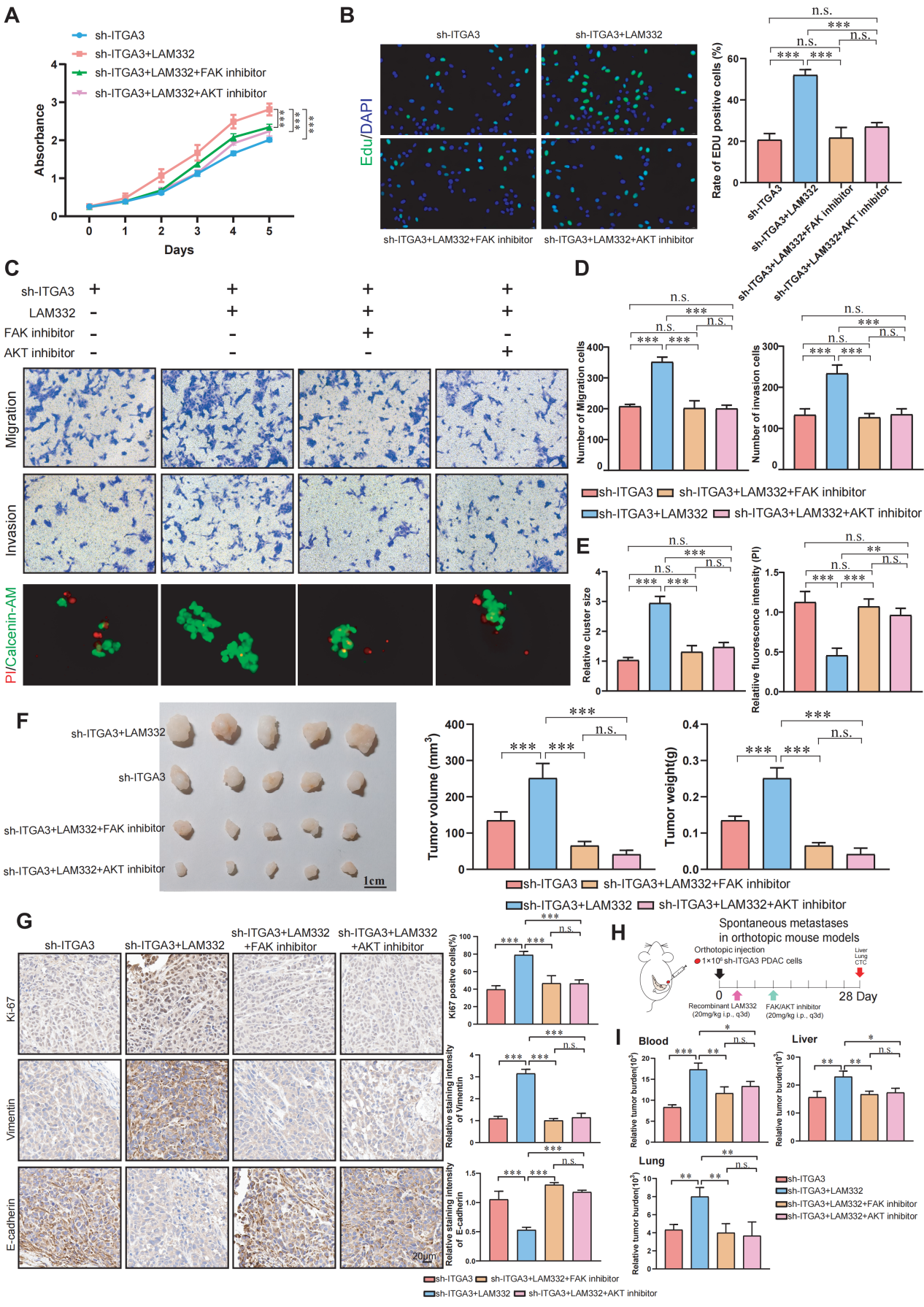
Supplementary Figure 6. CAFs-derived LAM332 enhances PDAC progression via ITGA3 on tumor cells. (A)(B)(C) CCK-8 and colony formation assays were conducted on PDAC cells. (D)(E)(F) Cell migration and invasion capacity were determined via a wound healing assay and a transwell assay. (G)(H)(I)(J) Clustering assays, live/dead viability assays and apoptosis assays were performed for cells after 24 hours of suspension culture. (K)Schematic diagram of the subcutaneous xenograft models. (L)(M)(N) Comparison of subcutaneous tumor volume and weight among the different groups. (O) H&E and IHC staining of Ki67, E-cadherin, and vimentin.


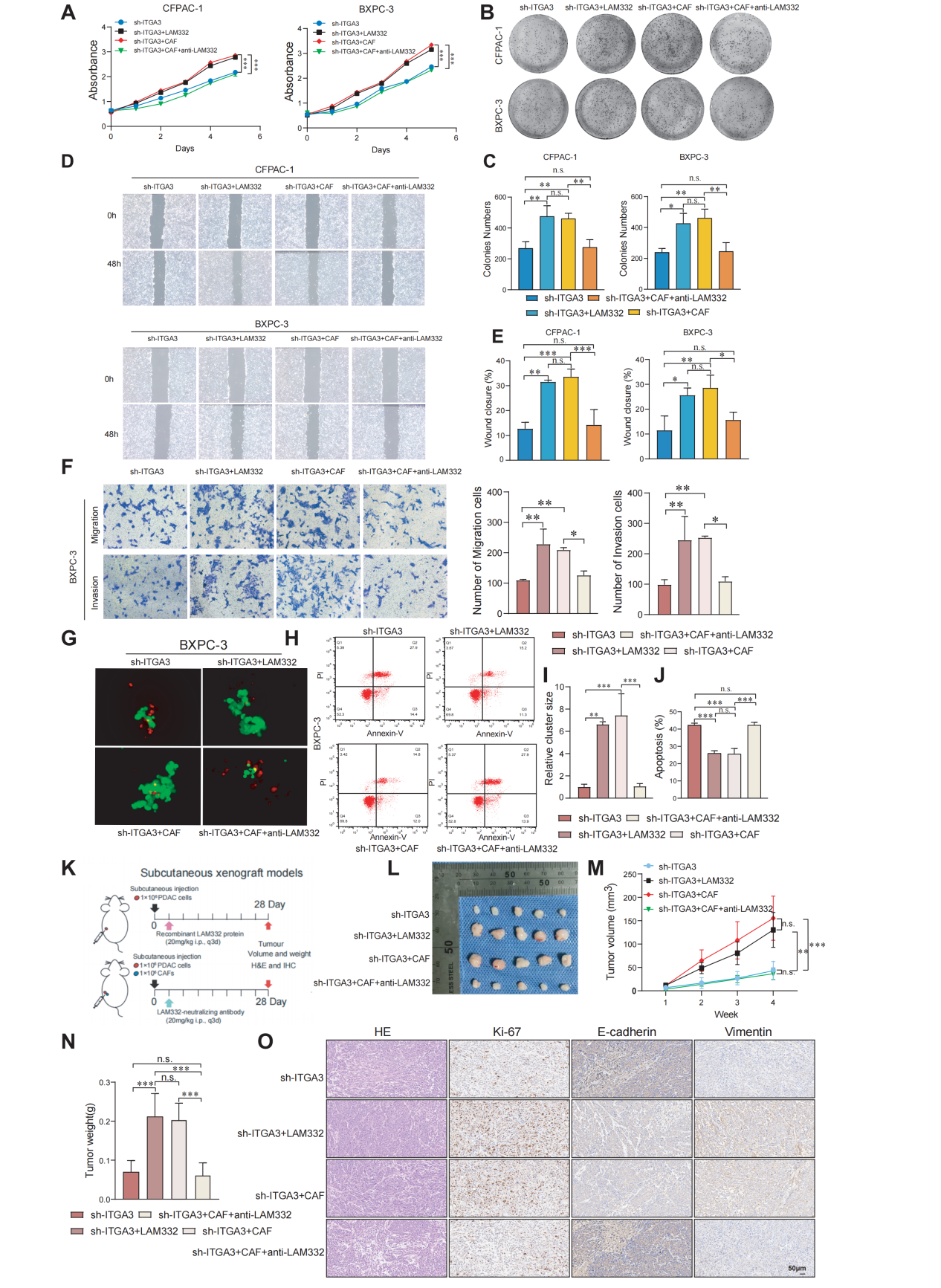

Supplement: Supplementary file 1 — Supplementary figures and tables [file 41419_2026_8642_MOESM1_ESM.docx]
