# Supplementary material for: CAFs-derived LAM332 promotes CTCs formation and survival via ITGA3 and contributes to the metastasis of pancreatic ductal adenocarcinoma
Source: Cell Death Dis. 2026 Mar 25;17(1):369. doi: 10.1038/s41419-026-08642-z (PMC13039154; doi:10.1038/s41419-026-08642-z)

Figure 3L

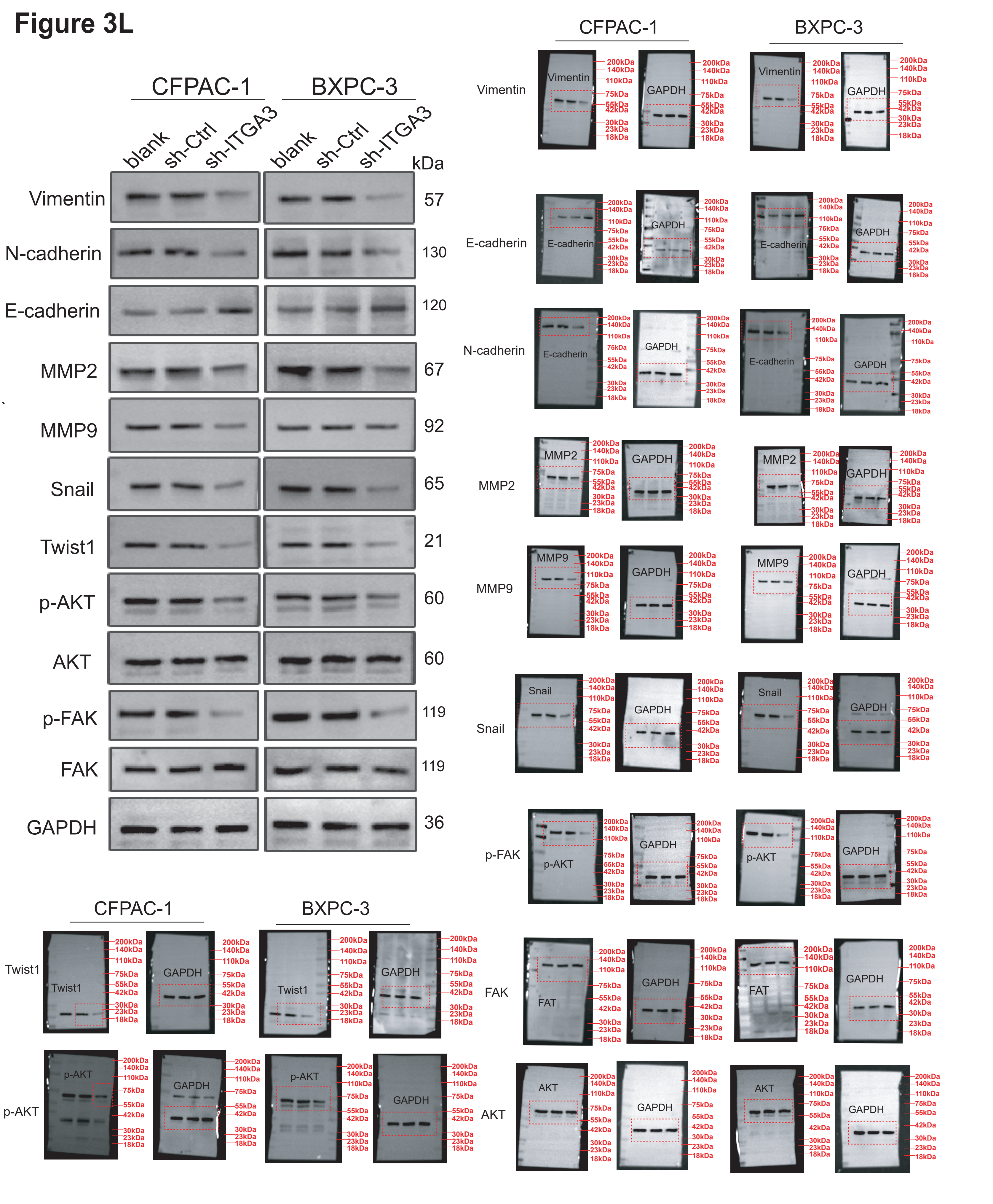

Figure 4G

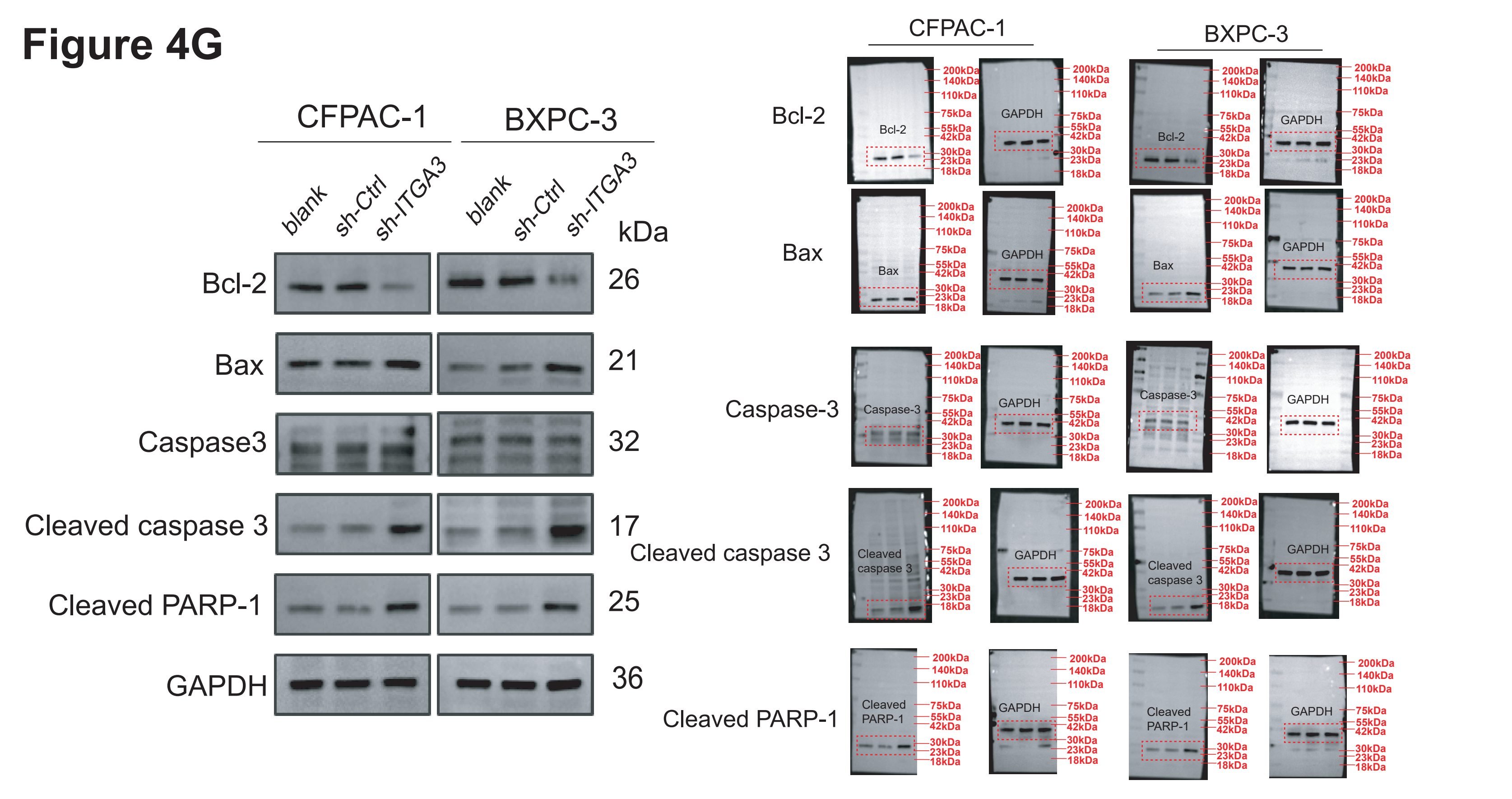

Figure 5H

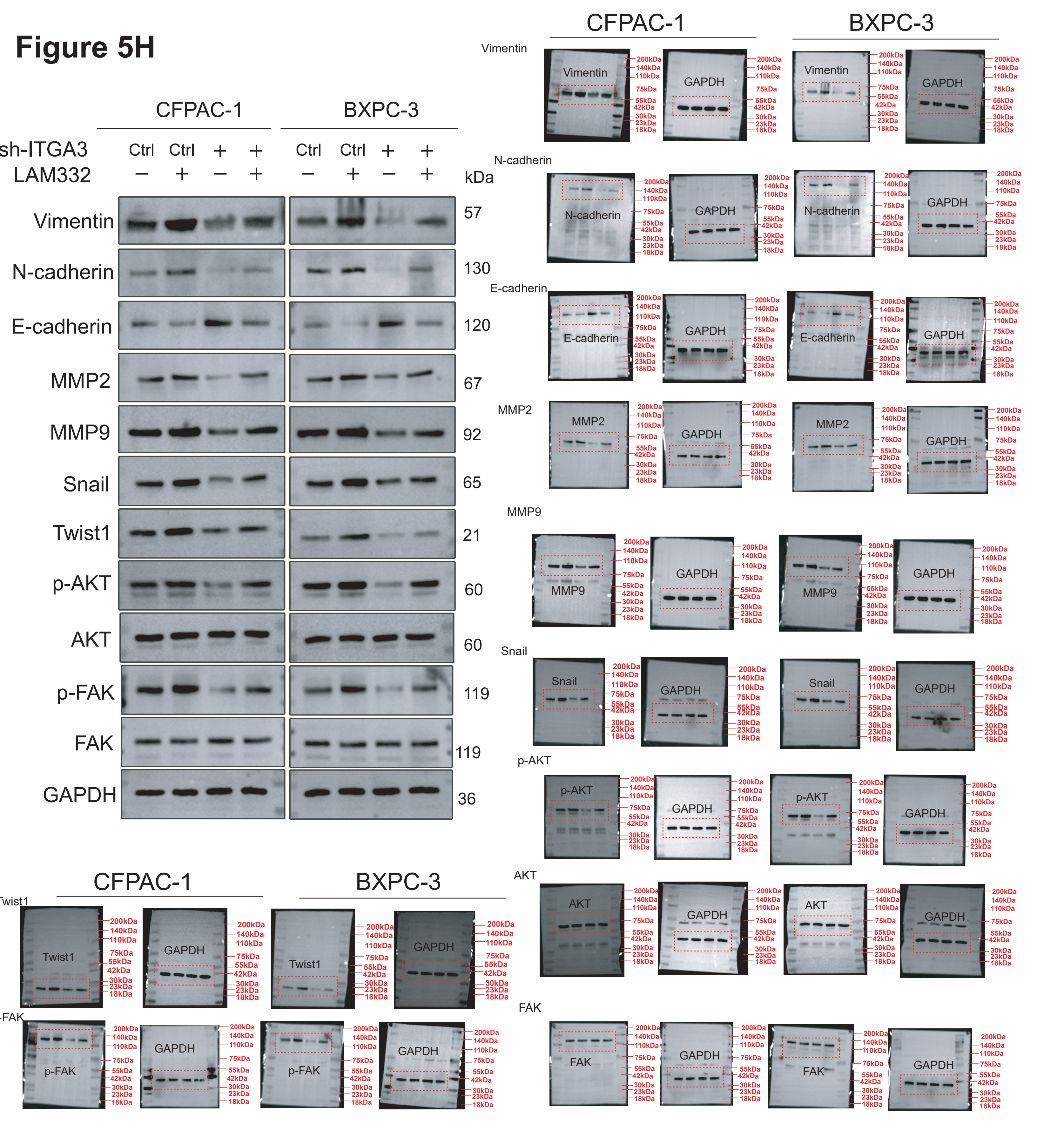

Figure6D

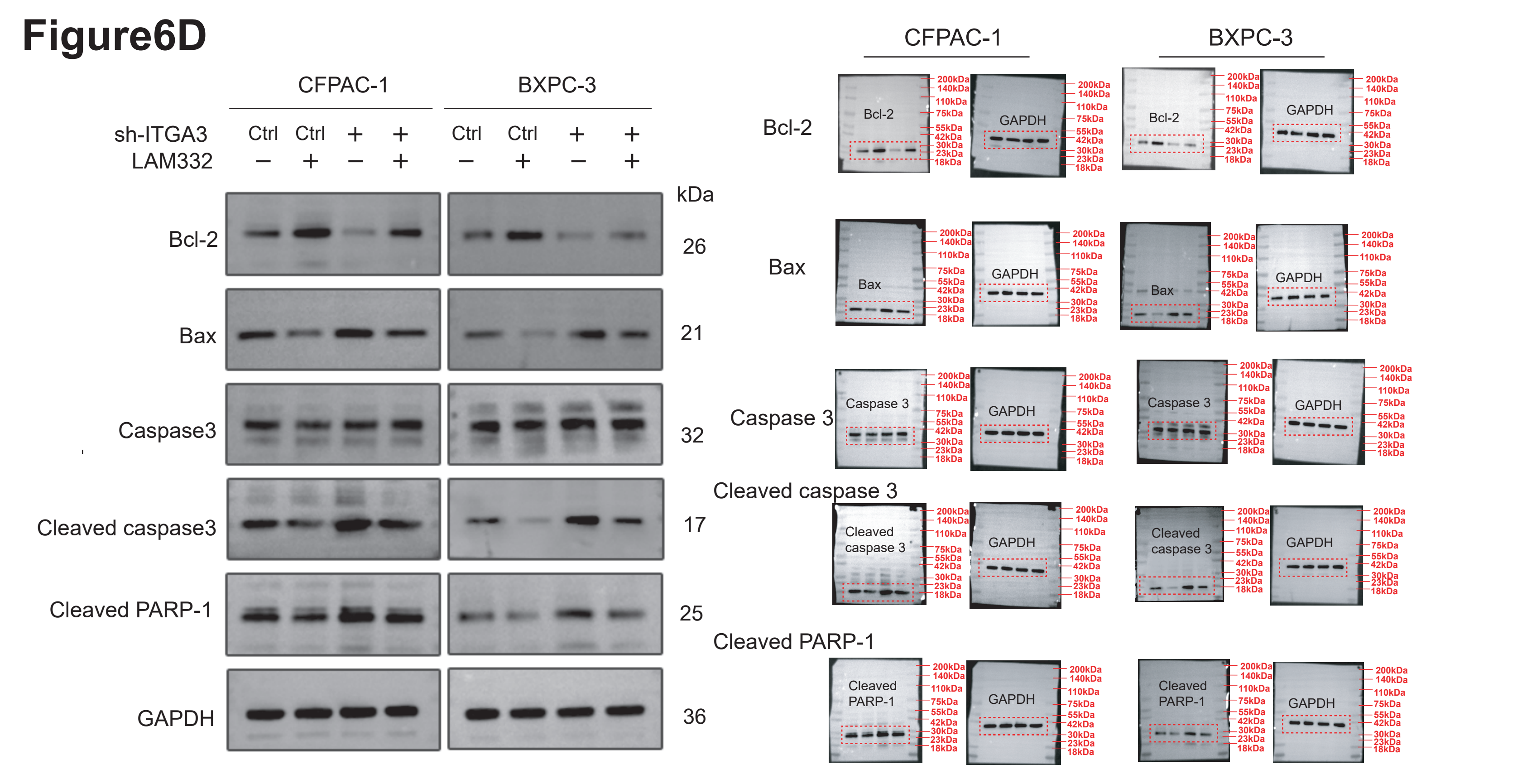

Figure 7B

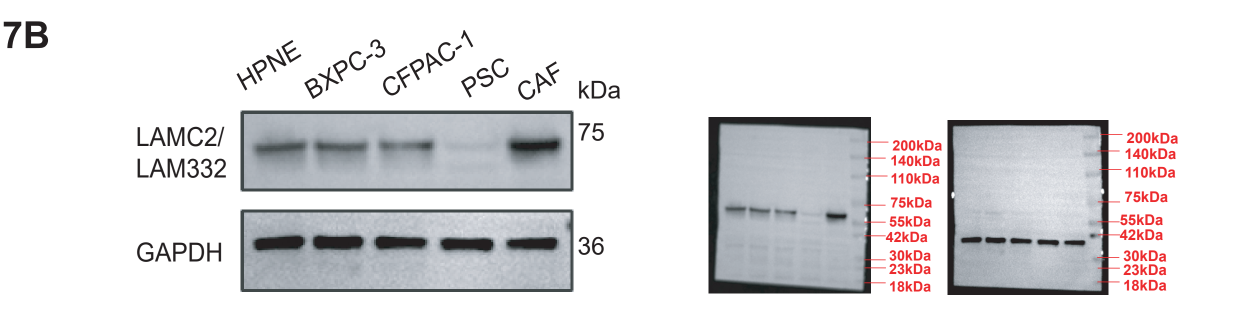

Figure 7I

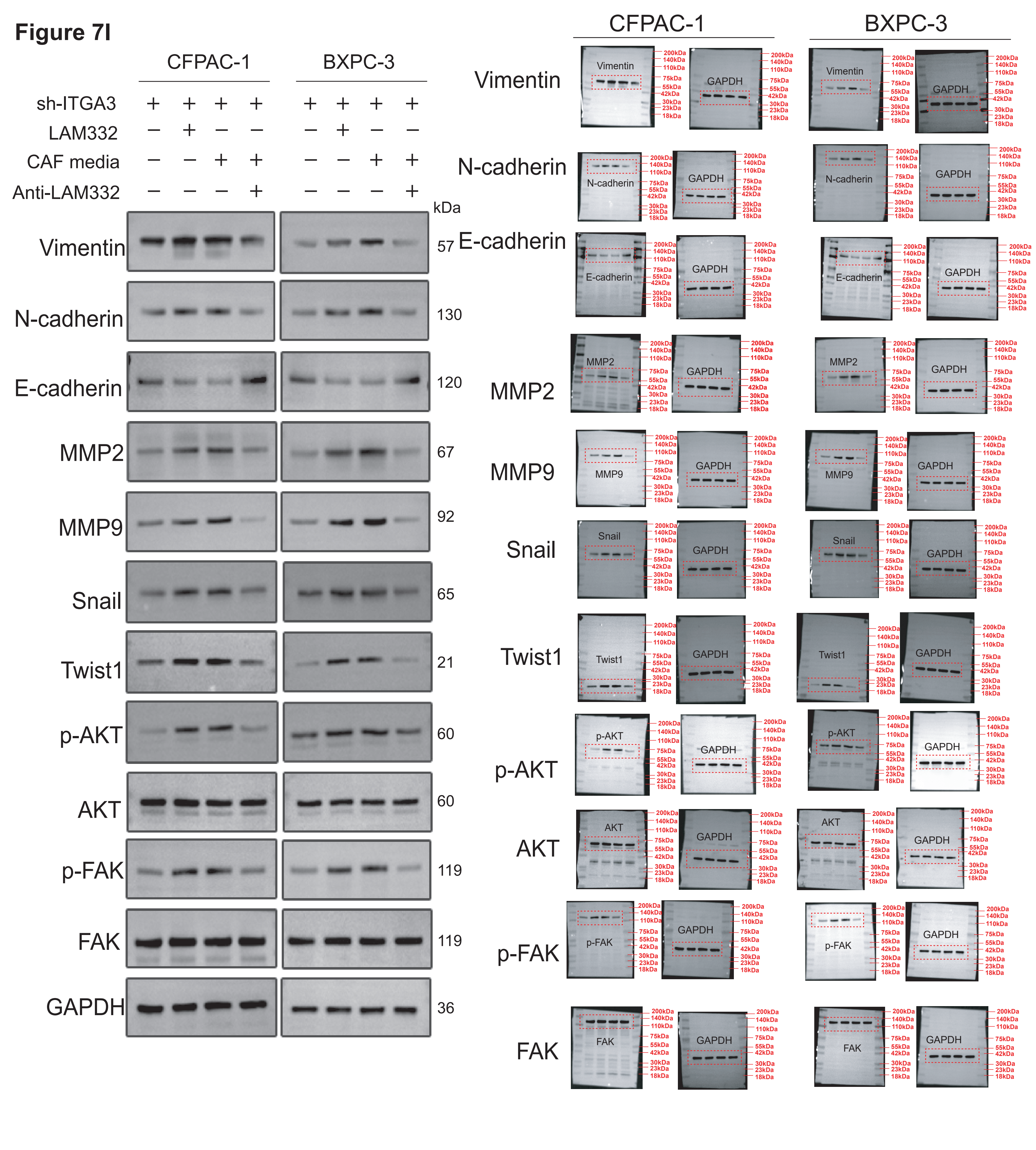

Figure 7L

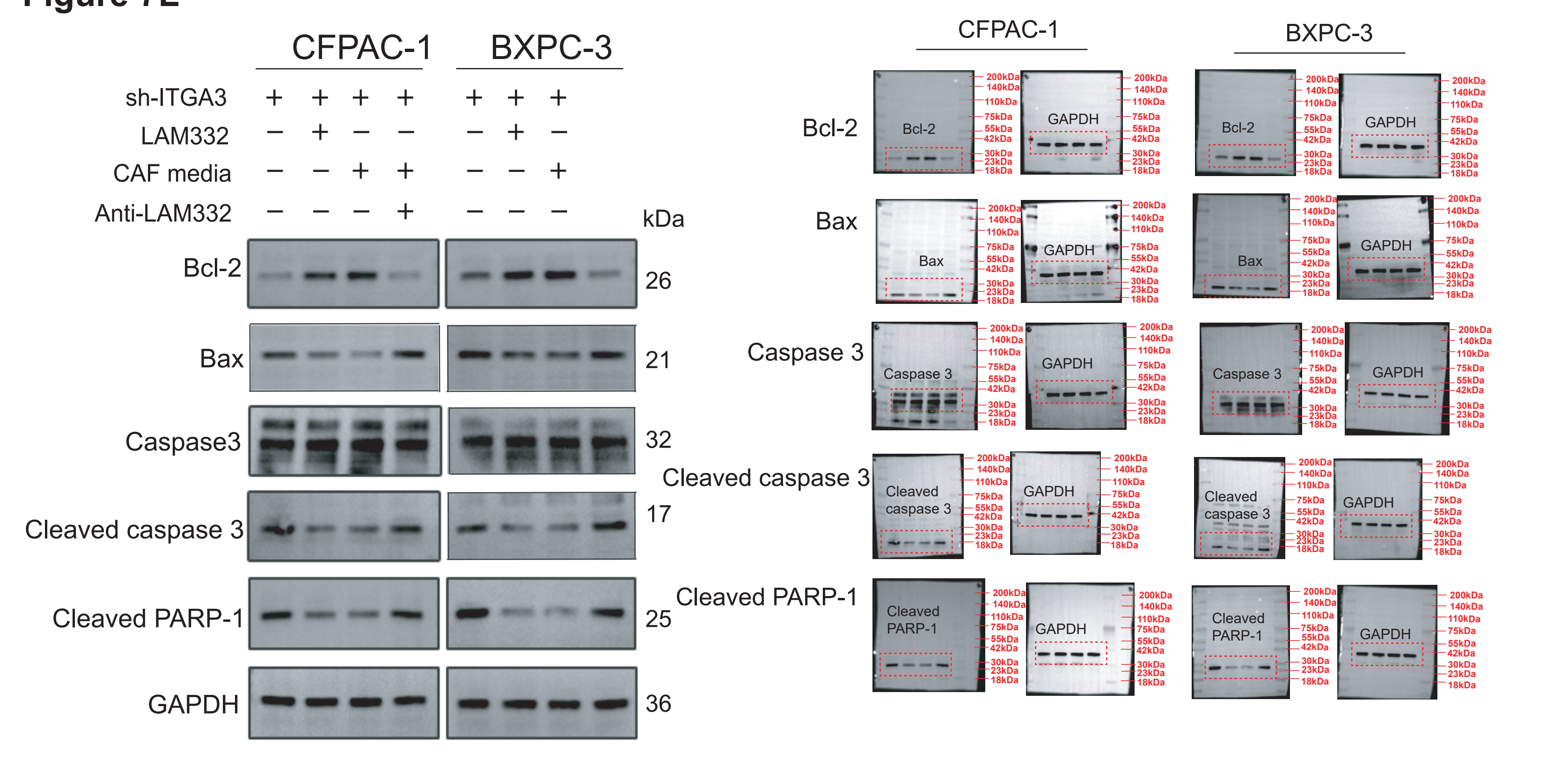

Supplementary Figure 2A

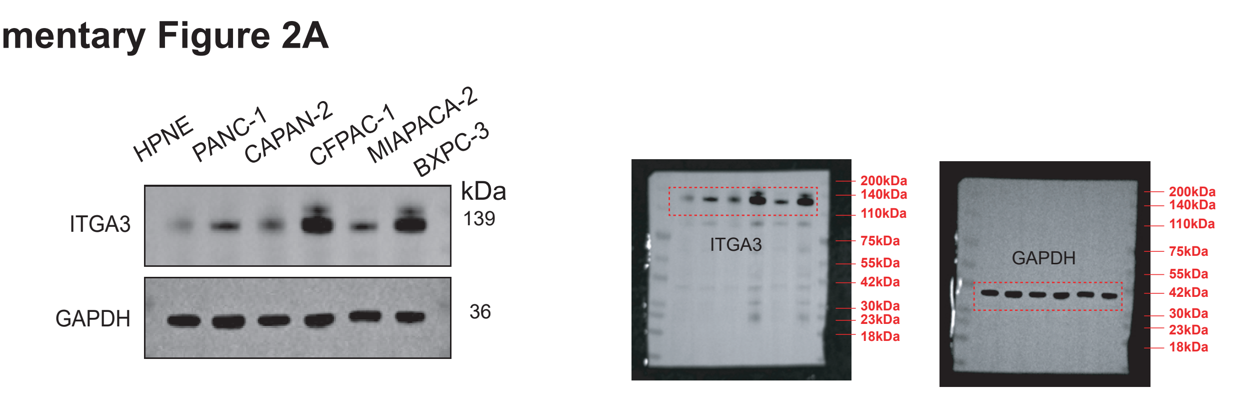

Supplementary Figure 2B

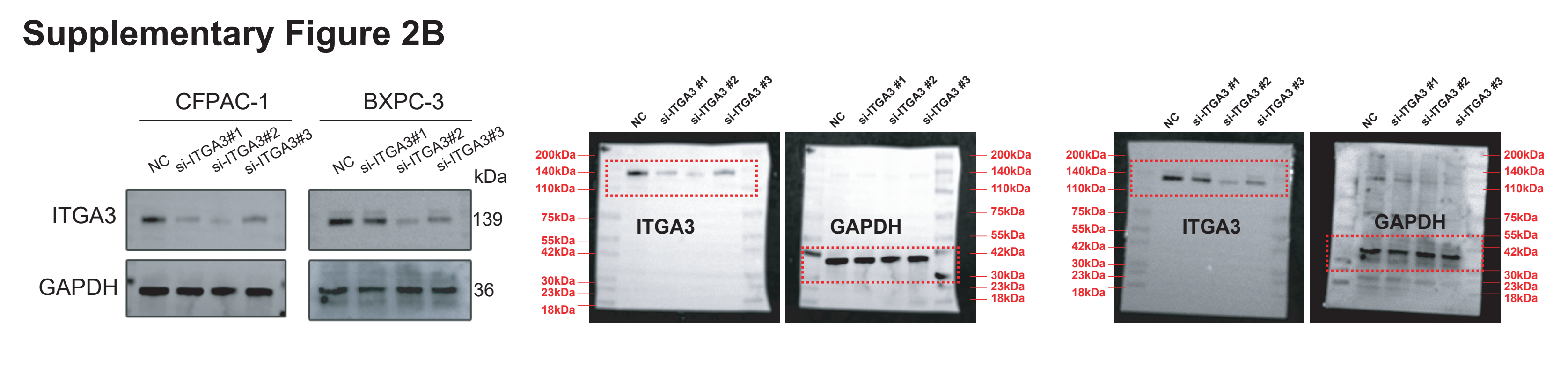

Supplementary Figure 2D

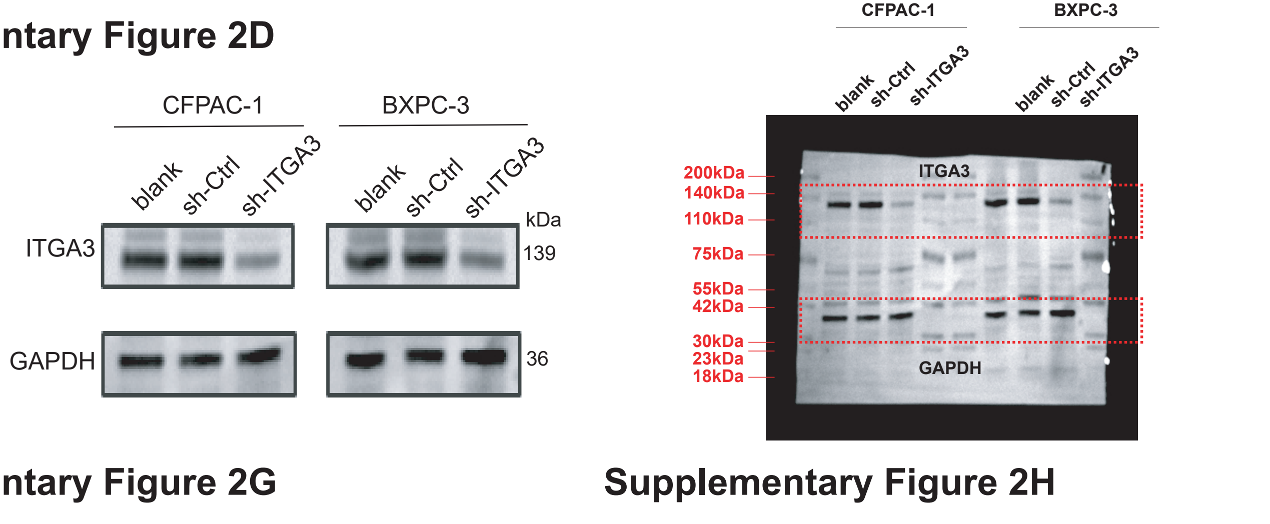

Supplementary Figure 2G

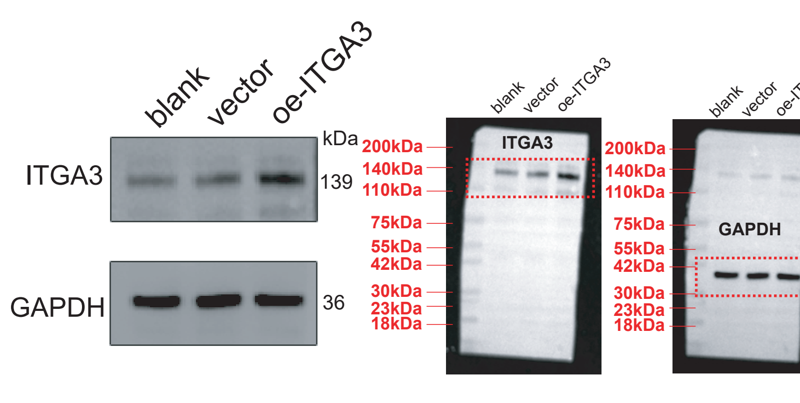

Supplementary Figure 2H

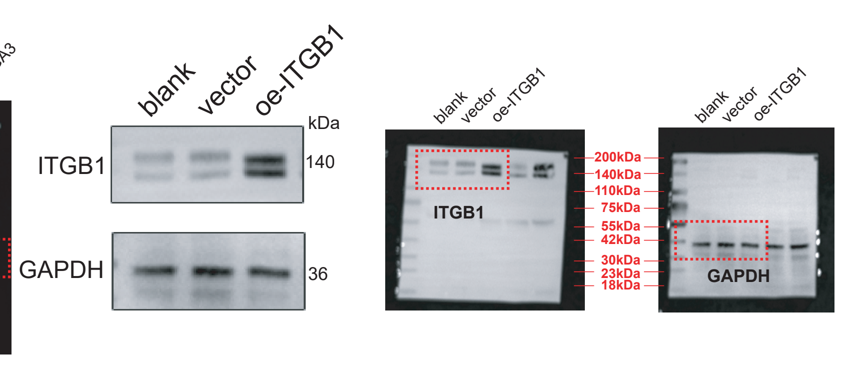

Supplement: Supplementary file 2 — Original Western blots. [file 41419_2026_8642_MOESM2_ESM.pdf]
